# Genome-Wide Association Study to Identify QTL for Carcass Traits in Korean Hanwoo Cattle

**DOI:** 10.3390/ani13172737

**Published:** 2023-08-28

**Authors:** Mohammad Zahangir Alam, Md Azizul Haque, Asif Iqbal, Yun-Mi Lee, Jae-Jung Ha, Shil Jin, Byoungho Park, Nam-Young Kim, Jeong Il Won, Jong-Joo Kim

**Affiliations:** 1 Department of Biotechnology, Yeungnam University, Gyeongsan 38541, Republic of Korea; mzalam-geb@sust.edu (M.Z.A.); azizul@ynu.ac.kr (M.A.H.); asif-gen@sust.edu (A.I.); ymlee@yu.ac.kr (Y.-M.L.); 2 Gyeongbuk Livestock Research Institute, Yeongju 36052, Republic of Korea; hjjggo@korea.kr; 3 Hanwoo Research Institute, National Institute of Animal Science, Pyeongchang 25340, Republic of Korea; jins21@korea.kr (S.J.); bhpark70@korea.kr (B.P.); rat1121@korea.kr (N.-Y.K.)

**Keywords:** candidate gene, carcass traits, genome-wide association study, Hanwoo, SNP

## Abstract

**Simple Summary:**

Genome-wide association study (GWAS) is a widely used approach to identify genetic variants and genomic regions associated with phenotypes. However, there are limited studies on GWAS that have focused on carcass traits in Korean Hanwoo using large sample sizes and employing residual or genomic estimated breeding value (GEBV) as response variables. Given these circumstances, our study aimed to reexamine GWAS in Hanwoo using a substantial population sample. We performed a simple single-marker regression analysis, utilizing the deregressed genomic estimated breeding value (DGEBV), GEBV, and residual values as response variables in genomic best linear unbiased prediction (GBLUP) and Bayes B methods. Our primary focus was on identifying common single nucleotide polymorphisms (SNPs) using both approaches, as this approach has been rarely reported. Therefore, the objective of this study was to uncover the genetic regions strongly associated with carcass traits in Hanwoo using a large cattle population. We identified 129 common SNPs using DGEBV and 118 common SNPs using GEBV, located on chromosomes 6, 13, and 14, demonstrating their significant associations with the studied carcass traits. The identification of these regions, along with the discovery of potential candidate genes, will contribute to a deeper understanding of the genetic and biological mechanisms underlying carcass traits in the Hanwoo population.

**Abstract:**

This study aimed to identify genetic associations with carcass traits in Hanwoo cattle using a genome-wide association study. A total of 9302 phenotypes were analyzed, and all animals were genotyped using the Illumina Bovine 50K v.3 SNP chip. Heritabilities for carcass weight (CWT), eye muscle area (EMA), backfat thickness (BF), and marbling score (MS) were estimated as 0.42, 0.36, 0.36, and 0.47, respectively, using the GBLUP model, and 0.47, 0.37, 0.36, and 0.42, respectively, using the Bayes B model. We identified 129 common SNPs using DGEBV and 118 common SNPs using GEBV on BTA6, BTA13, and BTA14, suggesting their potential association with the traits of interest. No common SNPs were found between the GBLUP and Bayes B methods when using residuals as a response variable in GWAS. The most promising candidate genes for CWT included *SLIT2*, *PACRGL*, *KCNIP4*, *RP1*, *XKR4*, *LYN*, *RPS20*, *MOS*, *FAM110B*, *UBXN2B*, *CYP7A1*, *SDCBP*, *NSMAF*, *TOX*, *CA8*, *LAP3*, *FAM184B*, and *NCAPG*. For EMA, the genes *IBSP*, *LAP3*, *FAM184B*, *LCORL*, *NCAPG*, *SLC30A9*, and *BEND4* demonstrated significance. Similarly, *CYP7B1*, *ARMC1*, *PDE7A*, and *CRH* were associated with BF, while *CTSZ*, *GNAS*, *VAPB*, and *RAB22A* were associated with MS. This finding offers valuable insights into genomic regions and molecular mechanisms influencing Hanwoo carcass traits, aiding efficient breeding strategies.

## 1. Introduction

The genetic improvement of cattle using DNA information began with the milestone of sequencing the genome of the Hereford cow (L1 Dominette 01449), which was deposited into free public databases in 2009 [1]. Subsequently, researchers worldwide embarked on sequencing the genomes of various cattle breeds, such as Angus, Brahman, Fleckvieh, Hanwoo, Holstein, Jersey, Japanese Black, Yanbian, Gir, Nelore, Limousin, and Norwegian Red, with the aim of identifying genetic variants associated with important phenotypic traits [2]. To facilitate these genetic studies, the first high-density genotyping SNP chip, the Illumina BovineSNP50 BeadChip, was developed by Illumina (USA) in 2007. This SNP chip consisted of 54,001 evenly spaced SNPs across the bovine genome and proved to possess useful minor allele frequencies (MAFs) in economically important cattle breeds [3]. With the availability of this high-density commercial SNP chip, genomic selection and genomic prediction were introduced into the routine genetic evaluation of dairy cattle [4]. These advancements in genomic technologies and data availability have opened new avenues for improving cattle breeding programs through more precise selection based on genetic information. The sequencing and genotyping efforts across diverse cattle breeds have provided valuable insights into the genetic basis of economically important traits, paving the way for enhanced genetic selection strategies and the continued progress in cattle improvement.

In recent times, cattle breeding has incorporated advanced molecular techniques and statistical approaches to identify causal mutations influencing traits. Most of the economic traits in cattle, known as quantitative trait loci (QTL), are polygenic in nature and controlled by multiple genes. Hanwoo, a beef cattle breed originally developed and bred in the Korean peninsula, has gained significant popularity among Korean consumers due to its exceptional marbled meat. This breed has been recognized as a valuable genetic resource for nearly a century. To enhance Hanwoo’s status as a leading marbled beef cattle breed, various breeding strategies and efforts have been implemented by both government and private agencies since the 1960s [5]. As a result, there has been remarkable improvement in both carcass quantity and quality in response to consumer demands. According to the Chung et al. [6] report, the average carcass weight of Hanwoo steers increased from 343 kg to 437 kg, and the marbling score increased from 3.6 to 5.6, between the years 2000 and 2016. Due to advancements in biotechnology-driven breeding and management systems, the beef cattle industry has become the second highest revenue-generating sector in the South Korean economy, following the swine industry. 

Animal geneticists are currently devoting significant efforts to identify the causal genes and mutations responsible for economic beef quality traits. In these studies, single nucleotide polymorphism (SNP) markers are commonly utilized in genome-wide association studies (GWASs) to pinpoint genes that influence specific traits. While SNPs located within the coding region of a gene typically have a direct impact on its phenotypic expression, the majority of SNPs are found in non-coding regions, including intergenic regions. Determining which SNPs affect gene function in these non-coding regions poses a significant challenge. Interestingly, intergenic SNPs have been observed to be significantly associated with phenotypes in GWAS, despite their location outside of coding regions [7]. GWAS offers an attractive approach for linking phenotype-associated SNPs to genes. Linkage disequilibrium (LD) serves to connect SNPs with nearby genes, although it has been observed that many phenotype-associated SNPs do not exhibit LD with any specific gene. To address this, a common practice is to map SNPs to the closest gene, with a suggested distance cutoff of 1 Mb upstream and downstream of the SNP in most GWAS studies [8]. This distance is deemed sufficient to identify nearby genes potentially influencing disease-associated genes in human studies.

As of 25 April 2023, significant progress has been made in the field of quantitative trait loci (QTL) mapping, leading to the identification of an impressive total of 193,898 QTL for 680 different base traits, 201 trait variants, and 258 eQTL genes within the cattle genome (CattleQTLdb, https://www.animalgenome.org/cgi-bin/QTLdb/BT/index accessed on 5 August 2023). Specifically, there have been 2138 QTL identified for carcass weight, 1653 for eye muscle area (Longissimus muscle area), 701 for backfat thickness, and 2436 QTL for marbling score. In the context of Hanwoo populations in Korea, genome-wide association studies have been conducted to map QTL affecting carcass weight, eye muscle area, backfat thickness, and marbling score. However, previous GWAS studies on Hanwoo carcass traits [9,10,11,12,13,14] have yielded inconsistent results, which can be attributed to variations in the sample size and methodological approaches employed in different studies. It is important to note that most of these studies used the residual value as the response variable in GWAS. Moreover, there is a limited number of GWAS studies with sample sizes exceeding ~3000 individuals specifically focused on carcass traits in Hanwoo. Therefore, there is a need to revisit GWAS in Hanwoo using a larger population sample to obtain more reliable and comprehensive results. In our study, we analyzed a population of over 7300 individuals, which represents a significant improvement in terms of sample size compared to previous studies. For the GWAS analysis, we employed a simple single-marker regression analysis using the deregressed genomic estimated breeding value (DGEBV), GEBV, and residual values as response variables. This approach allowed us to identify the SNP markers that have a significant impact on the studied traits. Importantly, we focused on the common SNPs identified by both the GBLUP and Bayes B methods, as this approach is not extensively explored in existing literature.

The significance of our study lies in the comprehensive and robust investigation of Hanwoo cattle, utilizing a large sample size and incorporating state-of-the-art GWAS methodologies. Through our research, we strive to bridge the knowledge gap in the understanding of carcass traits in Hanwoo populations and provide a solid foundation for future breeding programs. By unraveling the genomic regions that control these traits, we can facilitate the development of more effective breeding strategies to enhance economic considerations in the Hanwoo industry. Therefore, our objectives were to identify significant SNPs associated with these traits, explore the genetic architecture and biological relevance of these markers at the whole genome level, and elucidate the potential candidate genes involved in Hanwoo cattle.

## 2. Materials and Methods

### 2.1. Animal Phenotypes

Phenotypic data were collected from a mainland Hanwoo steers population comprising 9302 animals across various regions of Nonghyup livestock farms in Korea. The animals were born between the years 2014 and 2015 and raised for a period of 30 to 31 months under standard feeding and management practices outlined by Chung et al. [6]. Subsequently, they were slaughtered between 2017 and 2018. Phenotypic data related to carcass traits, namely, carcass weight (CWT), eye muscle area (EMA), backfat thickness (BF), and marbling score (MS), were recorded following the Korean carcass grading procedure established by the National Livestock Cooperatives Federation. Specifically, CWT measurements were obtained after 24 h of postmortem refrigeration. EMA was measured using a dot–grid technique on a cross-sectional slice between the 13th rib and the 1st lumbar vertebrae perpendicular to the vertebral column, where BF was also measured. The marbling score, on the other hand, was assessed visually using a categorical system consisting of 9 levels ranging from 1 (no marbling) to 9 (high marbling), as outlined in the Livestock Products Grading Guideline 2011.

### 2.2. Genotyping and Quality Control

A total of 9302 Hanwoo from the mainland were genotyped using the Illumina Bovine SNP50K v.3 BeadChip (Illumina Inc., San Diego, CA, USA), which contained 52,122 embedded SNPs. SNPs located on sex chromosomes and with unknown and duplicate positions were removed for further quality control (QC) procedures. Several QC thresholds were set to remove poor-quality SNPs for further analysis. SNPs were discarded from the analysis when the SNP call rate was less than 90%, individuals had a genotyping call rate less than 90%, and the minor allele frequency (MAF) was less than 1% (monomorphic). The genotype frequency significantly deviated (*p* < 0.001) from the Hardy–Weinberg Equilibrium (HWE). The identity-by-state (IBS) test was performed to determine if there were similar individuals or genotyping errors in the datasets. Pairs of individuals showing a similarity rate greater than 99% were considered either identical animals or indicative of genotyping errors. The entire QC process and IBS test were performed through the PLINK v1.9 toolset [15]. Furthermore, the missing alleles were imputed using Beagle v5.4 software [16]. After conducting the IBS and QC tests, a total of 41,496 SNPs and 8856 animals remained in the dataset. From this, we selected 7328 Hanwoo steers with available SNP genotype and phenotype information for further genome-wide association analysis of carcass traits.

### 2.3. Statistical Analysis

The statistical significance of the fixed factors and covariates were tested using ASReml-SA v4.2 [17] for fitting the factors into the animal model. The single-trait animal model was implemented for GBLUP as follows [18]:

(1) y=Xb+Zu+e

where y represents the vector of phenotypic records; b is the vector of fixed effects of farm location, birth year, birth season, and slaughter age as a covariate; u is the vector of random genetic additive effects; e is the vector of random residual effects; X and Z are incidence matrices related to fixed and random genetic additive effects, respectively. In matrix notation, the mixed model equation (MME) could be written as:

(2) $$\left[\begin{array}{cc} X^{\prime }X & X\prime Z\\ Z\prime X & Z^{\prime }Z+G^{-1}\alpha \end{array}\right]\left[\begin{array}{c} \hat{b}\\ \hat{a} \end{array}\right]=\left[\begin{array}{c} X\prime Y\\ Z\prime Y \end{array}\right]$$

where $\alpha =\sigma_{e}^{2}/\sigma_{g}^{2}$, $\sigma_{g}^{2}$ is the genetic variance, $\sigma_{e}^{2}$ is the error variance, and $G^{-1}$ is the inverse of the genomic relationship matrix (GRM).

The genomic relationship matrix (G) was built using the Genome-wide complex trait analysis (GCTA) tools developed by Yang et al. [19], which efficiently holds the genomic relationships between animals [18]. The following equation was used to make the G matrix based on marker allele frequencies:

(3) $$G=\frac{(M-P)({M-P)}^{\prime }}{2\sum_{i=1}^{n}P_{i}(1-P_{i})}$$

where the marker matrix M has dimensions of n × m, n is the number of individuals, and m is the number of markers used. The marker alleles M were coded as AA (homozygous for the first allele) = 1, AB (heterozygous) = 0, BB (homozygous for the second allele) = −1. The element of the P matrix was calculated using the formula $P_{i}=2(P_{i}-0.5)$, where $P_{i}$ represent the minor allele frequency of the marker at locus i. M−P represents the incidence matrix (Z) for markers.

#### 2.3.1. Breeding Value Estimation

The GEBV of an animal i was calculated after estimating the marker effects using the formula below:

(4) $$GEBV=\sum_{j=1}^{m}z_{ij}{ĝ}_{j}$$

where z_ij_ denotes the genotype of the individual i at marker loci j, ĝ_j_ represents the allele substitution effect at locus marker j, and m is the number of markers. 

The DGEBVs were obtained following the Garrick’s method [20] as follows:

(5) $$DGEBV=GEBV/r^{2}$$

Here, $r^{2}\;$is the reliability of GEBV.

#### 2.3.2. Regression of SNP Markers on Phenotype

For GWAS analysis, the de-regressed genomic estimated breeding value (DGEBV), genomic estimated breeding value (GEBV), and the residuals (obtained from a mixed-model analysis by ASReml-SA v4.2) were used to estimate by fitting the following single-marker regression analysis:

(6) $$y_{DGEBV}=1_{n}\mu +Zg+e$$

(7) $$y_{GEBV}=1_{n}\mu +Zg+e$$

(8) $$y_{residual}=1_{n}\mu +Zg+e$$

where $y_{DGEBV}$, $y_{GEBV}$, and $y_{residual}$ are the vectors of the DGEBV, GEBV, and residual, respectively; $1_{n}$ is a vector of 1 s; μ is the overall mean; Z is the design matrix allocating to the records of the marker; g denotes the marker effects; and e is the vector of random residuals or errors, which follows the standard normal distribution with 0 mean and error variance $\sigma_{e}^{2}.$ In this model, the marker effects are fitted as fixed effects. It is worth noting that g is a vector whose size equals the number of SNP marker alleles since we only estimated additive effects. In the additive model, the SNP genotypes were coded as 1, 0, and −1 for the AA, AB, and BB genotypes, respectively, indicating the allele substitution effect of B on allele A. 

The PLINK v1.9 toolset [15] estimates the SNP effects by regressing the residuals of each phenotype on additive effects of each SNP using the ordinary least square (OLS) method, and the *p*-value for the regression coefficient was estimated. If the SNP marker has a significant effect for the trait, then it can be assumed that the SNP was in a linkage disequilibrium (LD) state with an unobserved QTL. The null hypothesis (H_0_ = the marker had no effect) was set to test the effect of the marker SNP on the trait and alternative hypothesis vice versa. The significance of the test statistic *p*-value threshold was set at (−log_10_P = 4) for each SNP.

#### 2.3.3. GWAS by Bayes B Method

The genomic prediction methodology with Bayes B was performed using the GenSel v4.90 program [21]. The Bayes B method assumed most of the genetic markers have zero effect and that only a few loci contribute, with some genetic variance [22]. The Bayes B statistical model is as follows [23]:

(9) $$y=\mu +Xb+\sum_{i=1}^{i=41496}Z_{i}a_{i}+e$$

where y is the vector of phenotypes; μ is the overall mean; b is the vector of fixed and covariate effects; X is the incidence matrix of the fixed and covariate effects; $Z_{i}$ is a vector of genotypes of a fitted marker i that is coded as 10, 0, or −10; $a_{i}$ is a random substitution effect of the fitted marker i with its variance; and e is the vector of random residuals. 

In order to determine the marker effects, a mixture model was applied, i.e., a fraction of markers (probability π) with zero effect and (probability 1 − π) of markers with non-zero effects, which was used to predict GEBV. Then, the genetic variances of the markers with non-zero effects would have $\sigma_{ai}^{2}>0$ [23]. In this study, the π values were assumed to be 0.998.

The fixed effects and covariate for each trait were fitted under the GBLUP model. For the Bayes B analysis, the estimates of genetic and residual variances that were obtained from ASReml-SA analysis were used as prior values. A total of 41,000 iterations of Markov chain Monte Carlo (MCMC) were run for the analyses, after discarding the first 1000 iterations of the burn-in period, and each of 100 iterations was selected to calculate the posterior mean and variance for the marker effects. 

The GWAS by the Bayes B model using the GenSel v4.90 program [21] is conducted after estimating the breeding values with the aforementioned Bayes B formula. The equation for this analysis is as follows [24]:

(10) $$y_{DGEBV}=\mu +\sum_{i=1}^{n}Z_{i}a_{i}+e$$

(11) $$y_{GEBV}=\mu +\sum_{i=1}^{n}Z_{i}a_{i}+e$$

(12) $$y_{residual}=\mu +\sum_{i=1}^{n}Z_{i}a_{i}+e$$

where $y_{DGEBV}$, $y_{GEBV}$, and $y_{residual}$ are the vectors of the DGEBV, GEBV, and residual, respectively; μ is the overall mean; n is the number of SNPs; $Z_{i}$ is the genotype covariate of the ith SNP; $a_{i}$ is the allelic substitution effect of SNP_i_; and e is the vector of random residuals. 

The estimated variance components were used as prior information, and a total of 41,000 MCMC iterations were implemented, with 1000 discarded as burn-in. Genetic variance explained by SNPs was calculated using fixed windows comprising 20 consecutive SNP markers. The genetic variance of SNP windows was determined as the sum of each SNP’s variance, calculated as follows:

(13) $${2p}_{i}(1-p_{i})u_{i}^{2}$$

where $p_{i}$ denotes the minor allele frequency, and $u_{i}^{2}$ denotes the i^th^ estimated SNP marker effect.

### 2.4. Bioinformatics Analysis

#### 2.4.1. Identifying Overlapping QTL

QTL regions were determined based on the location of the significant SNPs. We retrieved the whole genome cattle (*Bos taurus*) QTL database (https://www.animalgenome.org/cgi-bin/QTLdb/BT/index) for searching QTL—release 48, accessed in November 2022. The available BED file with the QTL coordinates according to the cattle genome assembly (Bos_taurus_UMD_3.1.1) was used to check the QTL identified in our significant SNPs on respective chromosomes. The genomic positions and window that did not overlap with previously annotated QTL for meat and other carcass traits were considered as novel QTL affecting the trait.

#### 2.4.2. Finding Nearby Candidate Genes

Putative candidate genes within the SNP or genomic window regions and in the neighboring 1 Mb upstream and downstream regions of our mapped significant SNPs were identified based on the Bos_taurus_UMD_3.1.1 genome assembly, using four online resources, such as Biomart tools from Ensembl (http://asia.ensembl.org) accessed on 10 June 2023, NCBI (genome data viewer), Bovinemine V1.6 of the bovine genome database (www.bovinegenome.org) accessed on 10 June 2023, and BGVD (http://animal.nwsuaf.edu.cn/code/index.php/BosVar) accessed on 12 June 2023. All four online resources were cross-checked to accurately identify all nearby genes in the significant SNP locations.

#### 2.4.3. Functional Annotations

The functional roles of the identified genes located within and nearby significant SNPs associated with the four carcass traits, viz., CWT, EMA, BF and MS, were explored through published reports in PMC (NCBI database) journals and other literature surveys. We also found the functional roles of each gene from the online resource of human gene functions at GENECARDS (www.genecards.org) accessed on 15 June 2023. The candidate genes that seem to functionally relate to our desired traits of interest were assumed as promising candidate genes.

Significant GO (gene ontology) terms provide insight into the functional characteristics of annotated genes. The gene ontology analysis was carried out to explore attributes of the genes, including the biological processes (BPs), molecular functions (MFs), and cellular components (CCs) of the candidate genes near our significant SNPs using the PANTHER v14.1 (Protein Analysis Through Evolutionary Relationships) web-based tools available at https://pantherdb.org (accessed on 15 June 2023) [25] and the DAVID v6.8 bioinformatics resources for the functional annotation tool [26]. We also explore the protein class and molecular pathways associated with the candidate gene with PANTHER tools, thus providing better insight into the functional characteristics of the annotated genes. For both functional and pathway analysis, a statistically significant *p*-value threshold was set at *p* ≤ 0.05. Manhattan plots were drawn using the R package qqman [27].

## 3. Results and Discussion

### 3.1. Summary Statistics of the Phenotypes

The summary statistics for the carcass traits (CWT, EMA, BF, and MS) of a mainland Hanwoo population consisting of 9302 animals, including 7328 steers, are presented in Table 1. The mean carcass weight in the studied Hanwoo population was approximately 430 kg (445 kg for steers) at an average slaughter age of around 30 months. A previous study by Kwon et al. [28] examined the Hanwoo population over a three-year period from 2013 to 2015 and reported average CWT values of 428 kg for males and 336 kg for females at approximately 31 and 52 months of slaughter age, respectively. In our study, the MS was estimated to be around 5.83 (6.31 for steers) on a 1–9 scale, which was higher than the values reported by Kwon et al. (5.6 for males and 4.11 for females). Additionally, the backfat thickness was estimated to be 14.09 (14.53 for steers), and the eye muscle area was 94.16 (96.70 for steers) in our study, both of which showed higher estimates compared to the values reported by Kwon.

In another recent report by Haque et al. [29], the authors examined genomic selection methods for the same four carcass traits in Jeju Black cattle, using Hanwoo steers as a reference population at approximately 24 to 35 months of age. The recorded average values for CWT, EMA, BF, and MS were 445.00 kg, 96.70 cm^2^, 14.20 mm, and 5.99, respectively, which aligns closely with our steers data records. Additionally, in another recent report by Mehrban et al. [30], genomic selection methods were investigated for the four carcass traits in 5218 Hanwoo steers at approximately 24 months of age. The recorded average values for CWT, EMA, BT, and the transformed marbling score (lnMS) were 341.01 kg, 78.73 cm^2^, 8.60 mm, and 1.38, respectively. The lower marbling score observed in their study was a result of the data being transformed using the natural logarithm to lnMS after adding 1 to all records, as the original MS data exhibited skewness. The phenotypic data in Mehrban’s study were lower than our records, likely due to the lower slaughter age in their study. However, these differences can be attributed to the fact that the Hanwoo carcass yield and quality have improved in recent years due to the implementation of genetic improvement programs by the government and farmers.

### 3.2. Estimation of Variance Components and Heritability

The additive genetic variance and residual variance were estimated using the GBLUP and Bayes B models. The heritability (h^2^) values for CWT, EMA, BF, and MS were calculated from these variance estimations and are presented in Table 2. Our study reported h^2^ (GBLUP, Bayes B) for the Hanwoo population as follows: CWT (0.42, 0.47), EMA (0.36, 0.37), BF (0.36, 0.36), and MS (0.47, 0.42). These estimates significantly differ from the h^2^ estimates obtained in recent studies by Haque et al. [29], who reported h^2^ for CWT, EMA, BF, and MS using the GBLUP method as 0.30, 0.26, 0.26, and 0.34, respectively. Mehrban et al. [30] also estimated h^2^ for these four traits using Bayes C and Bayes L methods and obtained almost similar values. We rechecked the h^2^ values using a fivefold cross-validated dataset and obtained results consistent with our initial estimates (Supplementary Material Table S1).

Furthermore, Park et al. [31] reported h^2^ of 0.30, 0.42, 0.50, and 0.63 for CWT, EMA, BF, and MS, respectively. Another report on the estimation of genetic parameters for carcass traits in Hanwoo beef cattle under a national-scale breeding scheme, which included 72,969 animals from 2002 to 2013, showed h^2^ values of 0.28 ± 0.019 for CWT, 0.23 ± 0.020 for EMA, 0.20 ± 0.018 for BF, and 0.28 ± 0.021 for MS [32]. The h^2^ estimates for most carcass traits exhibited significant variation, which could be attributed to factors such as breed differences, population structure, and environmental influences, among others. Additional factors that may contribute to variation in heritability estimates include the methods of estimation, effects in the statistical models, the number of observations, measurement errors, and factors related to sex and management [33]. In Hanwoo cattle, MS is consistently identified as the highest heritable trait among the four carcass traits, except in the study by Kim et al. [34], where it falls within the range of moderate h^2^. Our estimated h^2^ for CWT, EMA, and MS in Hanwoo cattle align closely with those previously reported by Koh et al. [35]. These findings suggest that meat quality traits in Hanwoo cattle are predominantly influenced by environmental effects, and selection for these traits would result in a moderate rate of genetic improvement.

Comparatively, in Japanese Wagyu cattle, Oyama [36] reported h^2^ of 0.46 for CWT, 0.49 for EMA, 0.32 for BF, and 0.21 for MS. Another report by Munim et al. [37] estimated h^2^ for Wagyu carcass traits within the range of 0.50 to 0.54. The h^2^ of Angus carcass traits were 0.40, 0.33, 0.34, and 0.45 for CWT, EMA, BF, and MS, respectively [38], which closely align with the Hanwoo traits estimated using the GBLUP method. The highest h^2^ values for MS were reported in the Simmental [39] and Shorthorn [40] breeds, which were 0.87 and 0.88, respectively.

In addition, Rolf et al. [39] conducted a study on h^2^ within and across multi-breed cattle populations using GBLUP and Bayesian models. They reported the highest h^2^ for backfat thickness in the Limousine breed (0.94). According to their reports, the four Bayesian models (Bayes Cπ, C0, A, and B95) estimated h^2^ values for CWT ranging from 0.48 to 0.59, ribeye area (REA) ranging from 0.32 to 0.36, fat thickness (FT) ranging from 0.06 to 0.27, and MS ranging from 0.62 to 0.72. Notably, larger differences in h^2^ values were observed within breeds. Several studies [4,41] have demonstrated a strong relationship between the accuracy of genomic prediction and the h^2^ of traits. It has been consistently observed in real data and simulation studies that higher h^2^ is associated with increased accuracy of genomic prediction [42,43]. However, all four carcass traits examined in the present study on Hanwoo cattle fall within the moderate range of heritability. This suggests that there are possibilities for future improvement through genomic selection.

### 3.3. GWAS Results for Carcass Traits

For the GWAS analysis, a simple single-marker regression analysis was performed using the de-regressed genomic estimated breeding value (DGEBV), GEBV, and residual values as response variables. This analysis allowed us to identify the SNP markers that affect the studied traits. Our focus was primarily on the common SNPs that were identified by both the GBLUP and Bayes B methods. In total, we detected 129 common SNPs using the DGEBV and 118 common SNPs using the GEBV on chromosome 6, 13, and 14, indicating their associations with carcass traits in the Hanwoo population under study. It is worth noting that no common SNPs were found between the GBLUP and Bayes B methods when using residuals as a response variable in the GWAS. The reasons behind this discrepancy could be attributed to the complex interplay of various factors influencing the genetic architecture and the specific statistical performance of the GBLUP and Bayes B methods. These factors may include the inherent characteristics of the traits, the modeling assumptions of the methods, and the noise introduced by using residuals as response variables. Further investigation and analysis would be needed to fully understand the reasons behind this discrepancy and to determine the potential biological or statistical factors contributing to the observed differences in SNP associations between the two methods when using residuals as the response variables. The detailed summary of the GWAS results, including significant SNP IDs for the four traits, SNP positions on the respective BTAs, effects on the traits, *p*-values, and nearby candidate genes with their type of consequence, is presented in Table 3 and Table 4, as well as Supplementary Material Tables S2 and S3. Within Table 3 and Table 4, we have focused on the top ten most significant SNP IDs based on their *p*-values for each trait, accompanied by their relevant information. Additional significant SNP IDs are detailed in Supplementary Material Tables S2 and S3. In Table 3, for the MS, only one common significant SNP is observed in both the GBLUP and Bayes B methods when DGEBV is utilized as the response variable. As for Table 4, no common significant SNPs were identified in either the GBLUP or Bayes B methods when GEBV is used as the response variable in BF and MS. To visualize the genome-wide distribution of significant SNPs, we generated Manhattan plots for carcass traits. The level of significance was represented as the negative logarithm base 10 (−log_10_) of each SNP’s *p*-value. Additionally, we depicted Q-Q plots to illustrate the observed versus expected *p*-values (−log_10_P) for each trait in the GBLUP method (Supplementary Material Figures S1–S4). These results are further described as follows.

#### 3.3.1. Carcass Weight (CWT)

**Figure 1:**
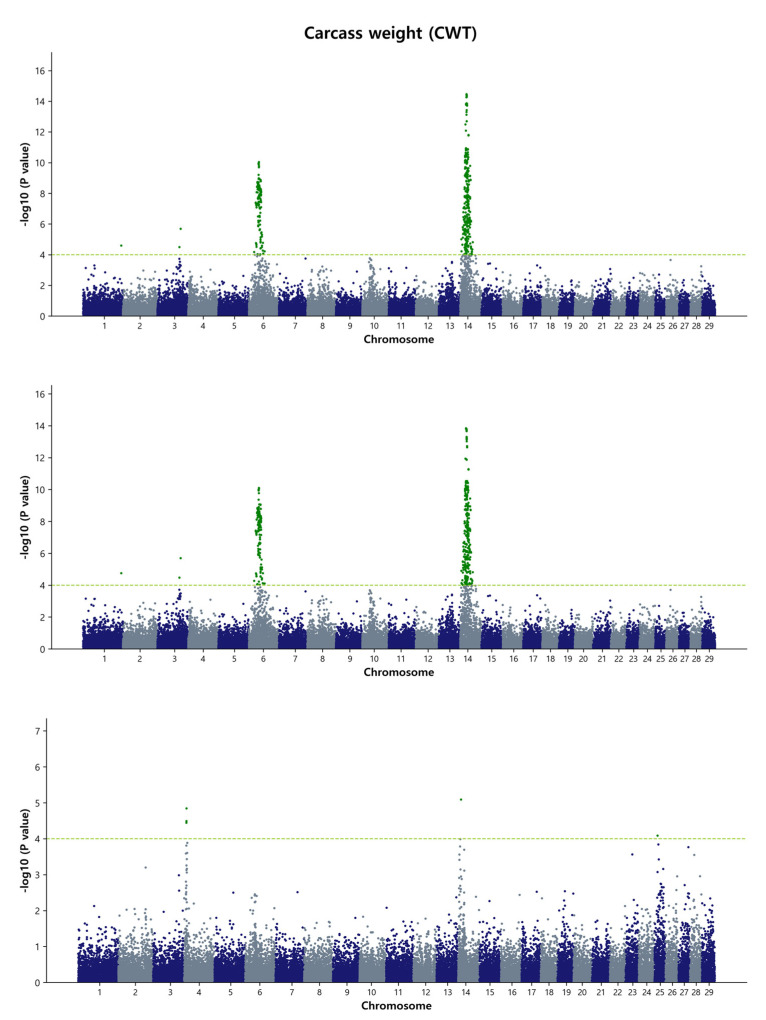
Manhattan plots of genome-wide −log_10_ (*p*-values) for carcass weight in Hanwoo cattle using DGEBV (**upper panel**), GEBV (**middle panel**), and residual (**lower panel**) as predictors in the GBLUP method. The *X*-axis represents the 29 *Bos taurus* autosomes, and the *Y*-axis represents the −log_10_P values. The horizontal dashed line in yellow–green indicates the significance threshold at *p* = 1 × 10^−4^ (4.00).

In the present study, the GWAS detected a total of 51 significant common SNPs associated with carcass weight when using the de-regressed genomic estimated breeding value (DGEBV) as a response variable. Additionally, 63 significant common SNPs were identified using GEBV as a response variable. For carcass weight in Hanwoo, two major chromosome regions were identified, 41.18 Mb to 41.98 Mb (0.8 Mb) on BTA6 and 24.14 Mb to 26.99 Mb (2.85 Mb) on BTA14, when using DGEBV as the response variable. However, when GEBVs were used without deregression, a novel region of 38.37 Mb to 38.99 Mb (0.62 Mb) on BTA6 was detected, while the position on BTA14 remained similar to that observed with DGEBV as the response variable (Figure 1 and Figure S5). The most significant SNPs (top five) based on the *p*-value threshold were rs41725159, *p* = 1.5 × 10^−13^; rs210258477, *p* = 1.63 × 10^−13^; rs42646659, *p* = 1.68 × 10^−13^; rs110092040, *p* = 1.68 × 10^−13^; and rs41725162, *p* = 1.72 × 10^−13^ for carcass weight, located on BTA14 at the position of 24.49 Mb to 26.62 Mb. Among these SNP markers, two SNPs, BTB-01143580 (rs42303720) and Hapmap30932-BTC-011225 (rs41724536), were previously identified in two other Hanwoo GWAS studies, affecting carcass weight on BTA14 (24.3 Mb and 24.8 Mb regions) [9,44]. In the cattle QTL database, a total of 595 QTL affecting carcass weight were identified, and our data revealed the overlap of at least 4 QTL on BTA6 (ID: 24623, 24634, 24635, and 24637) and 10 QTL on BTA14 (ID: 157040, 157041, 157042, 157043, 157044, 157045, 160768, 160769, 160770, and 160771) with the regions we detected.

#### 3.3.2. Eye Muscle Area (EMA)

**Figure 2:**
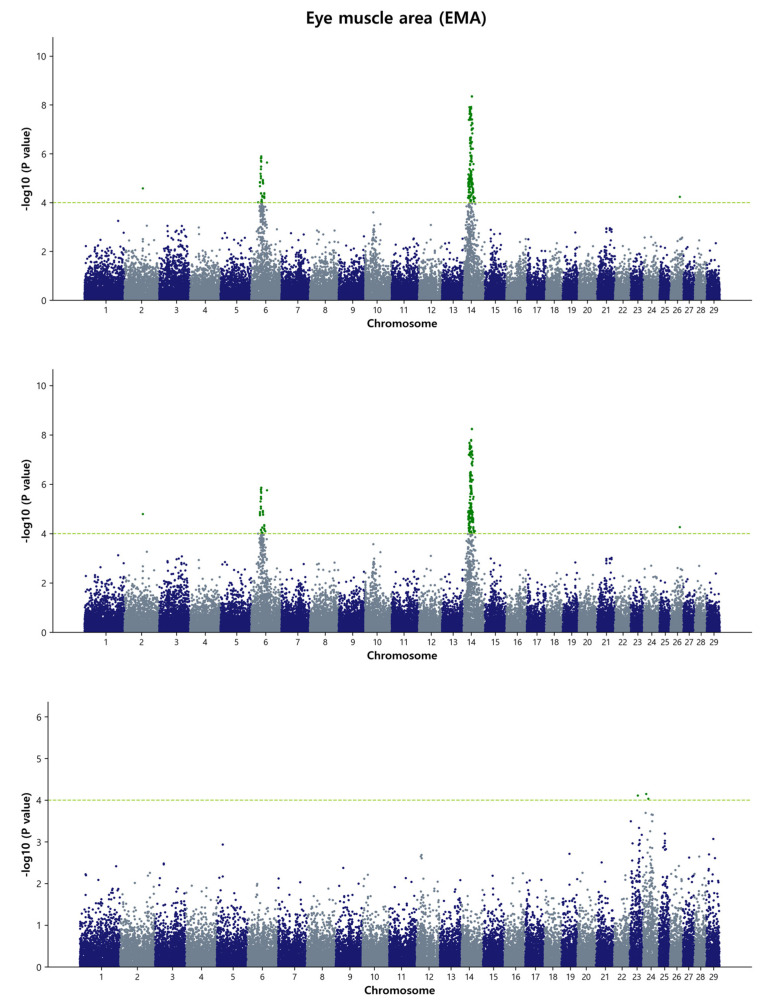
Manhattan plots of genome-wide −log_10_ (*p*-values) for eye muscle area in Hanwoo cattle using DGEBV (**upper panel**), GEBV (**middle panel**), and residual (**lower panel**) as predictors in the GBLUP method. The *X*-axis represents the 29 *Bos taurus* autosomes, and the *Y*-axis represents the −log_10_P values. The horizontal dashed line in yellow–green indicates the significance threshold at *p* = 1 × 10^−4^ (4.00).

For the eye muscle area, the GWAS identified three major chromosomal regions in Hanwoo: a region ranging from 38.37 Mb to 38.99 Mb (0.62 Mb) and 62.61 Mb on BTA6, and a region ranging from 24.14 Mb to 26.99 Mb (2.85 Mb) on BTA14. Both the DGEBV and GEBV models identified a total of 56 and 55 common SNPs, respectively. Additionally, a new region ranging from 38.46 Mb to 38.99 Mb (0.53 Mb) on BTA6 was identified when GEBV was used as the response variable. Among the reported QTL affecting the Longissimus muscle area trait in the cattle QTLdb database, 70 out of 431 QTL were found. Among these, 40 QTL were located on BTA6 and 30 QTL on BTA14. The most significant SNPs (top 5) based on *p*-value thresholds were rs798880362, *p* = 4.76 × 10^−9^; rs467005433, *p* = 5.07 × 10^−9^; rs432843769, *p* = 3.01 × 10^−8^; and rs110834363, *p* = 4.92 × 10^−8^ located on BTA6 at the 38.53–38.93 Mb region, and rs210258477, *p* = 6.02 × 10^−8^ on BTA14 at the 24.49 Mb region (Figure 2 and Figure S6). In a previous study, no SNP was detected for the EMA, but it was speculated that CWT is genetically correlated with the EMA, and a large number of genes with small effects are scattered across the whole genome [9]. Our study identified the same genetic region for both EMA and CWT using the Bayes B and GBLUP methods on BTA14. Furthermore, we found that one significant QTL (ID: 10968) on BTA14, ranging from 25.06 Mb to 31.87 Mb, overlapped with our study.

#### 3.3.3. Backfat Thickness (BF)

**Figure 3:**
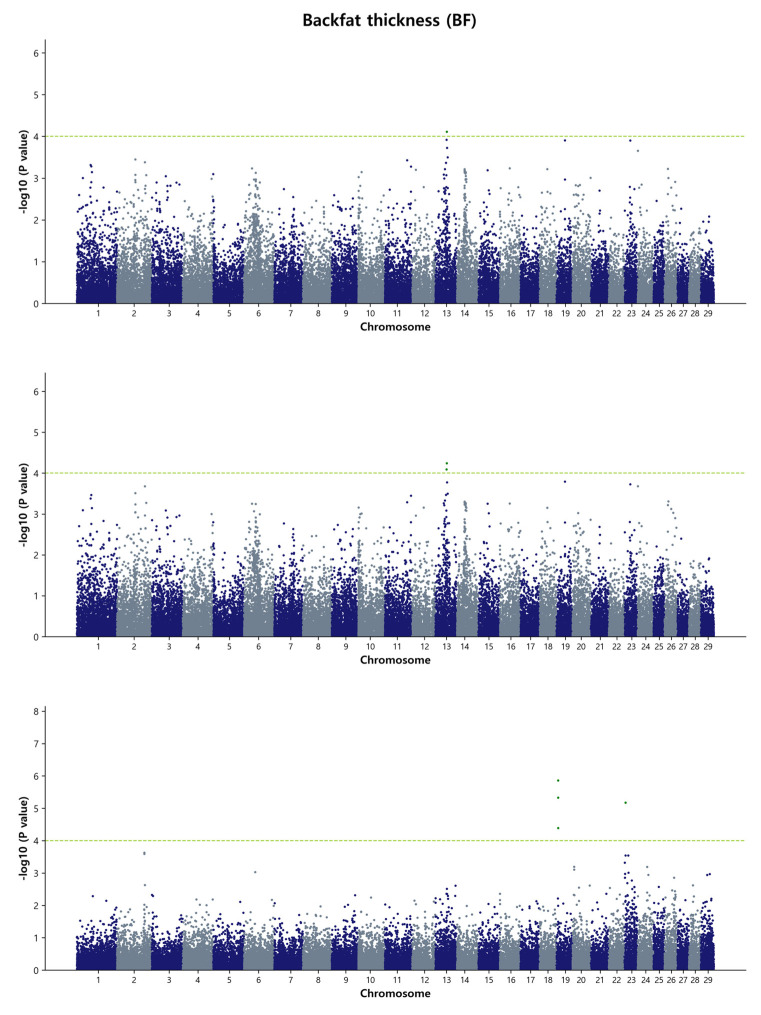
Manhattan plots of genome-wide −log_10_ (*p*-values) for backfat thickness in Hanwoo cattle using DGEBV (**upper panel**), GEBV (**middle panel**), and residual (**lower panel**) as predictors in the GBLUP method. The *X*-axis represents the 29 *Bos taurus* autosomes, and the *Y*-axis represents the −log_10_P values. The horizontal dashed line in yellow–green indicates the significance threshold at *p* = 1 × 10^−4^ (4.00).

The GWAS for BF in Hanwoo identified 21 significant SNPs on BTA14, specifically, in the region spanning 31.01 Mb to 31.98 Mb (0.97 Mb), using single-marker regression analysis (Figure 3). In the Bayes B model, the same region on BTA14 was detected, with 34 SNPs contributing to the analysis (Supplementary Material Figure S7). This region was found to account for approximately 2.6% of the genetic variance responsible for BF in Hanwoo, as estimated by the de-regressed breeding values. Among the top SNPs associated with BF, the five most significant ones were rs109546980, *p* = 3.49 × 10^−8^; rs41619147, *p* = 3.73 × 10^−8^; rs110366862, *p* = 6.16 × 10^−8^; rs41639002, *p* = 6.50 × 10^−8^; and rs42743206, *p* = 6.62 × 10^−8^. These SNPs are located on BTA4 and were detected in the Bayes B model, contributing to more than 1 % genetic variance in the Bayes B model, but did not reach the significance threshold in the GBLUP model. The cattle database indicated that at least 54 QTL may be associated with fat thickness. Interestingly, one QTL (ID: 10971) located at 31.74 Mb on BTA14 overlapped with the region we reported, providing further support for its potential role in BF in Hanwoo cattle.

#### 3.3.4. Marbling Score (MS)

**Figure 4:**
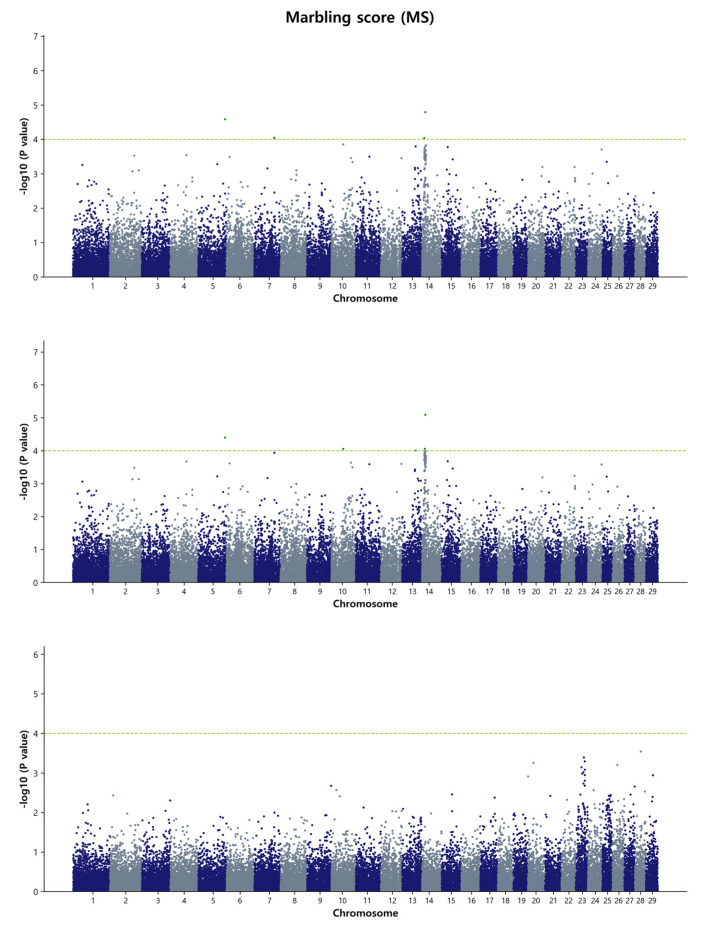
Manhattan plots of genome-wide −log_10_ (*p*-values) for marbling score in Hanwoo cattle using DGEBV (**upper panel**), GEBV (**middle panel**), and residual (**lower panel**) as predictors in the GBLUP method. The X-axis represents the 29 *Bos taurus* autosomes, and the Y-axis represents the −log_10_P values. The horizontal dashed line in yellow–green indicates the significance threshold at *p* = 1 × 10^−4^ (4.00).

For the marbling score, the GWAS identified a single common SNP (ARS-BFGL-NGS-119375, *p* = 8.63 × 10^−5^) located on chromosome region BTA13, which was detected by both the GBLUP and Bayes B methods (Figure 4 and Figure S8). The Bayes B method also detected three SNP windows on BTA5, BTA7, and BTA13 that collectively accounted for over 1% of the genetic variation in the MS. Notably, a specific chromosomal window in the region of 58.1 Mb on BTA13 accounted for more than 1.14% of the genetic variation for this trait. While the recent cattle QTL database identified eight QTL regions potentially associated with the trait, our data did not overlap or identify any previously reported associations in this specific region for the MS trait. Therefore, this chromosomal location can be considered a novel region potentially influencing the marbling score trait.

### 3.4. Candidate Gene

GWAS is a valuable tool for mapping phenotype-associated SNPs to genes or identifying variants within genes that control traits. One of the main challenges in GWAS is mapping SNPs to specific genes. While SNPs located in exons directly affect transcripts and traits, the majority of SNPs are found in non-coding regions (introns), making it difficult to determine their gene associations. Candidate genes, located near regions of association, can influence the expression of complex phenotypes due to their known biological and physiological properties. To assign SNPs to genes, it is common practice to use a distance cutoff. Various distance cutoffs have been used in previous GWAS studies to identify causal genes. Brodie et al. [8] suggested a justified upstream and downstream cutoff of 750 Mbps to map a SNP to a gene, which yielded statistically significant results. In some cases, SNPs located further away from genes, up to 2 Mbps, have also been observed. Considering the findings from previous GWAS studies, we extended our search for nearby candidate genes up to 1 Mbps in both directions from the QTL region. In our study, we focused on common SNPs identified by both the single-marker regression and Bayes B methods within a specific SNP window (Start~end). The most significant SNPs were found on BTA6 and BTA14 for the Hanwoo carcass traits (Table 3 and Table 4, Tables S2 and S3). Previous studies have considered BTA14 as a major source of genetic variation for beef carcass traits, including BTA 2, 3, 6, 7, 9, 11, 13, 14, 16, 17, 18, 20, 23, 26, 28, and 29 [44,45]. However, in our study, the most significant SNPs for carcass weight were distributed over BTA6 (from 41.18 Mb to 41.98 Mb and 38.37 Mb to 38.99 Mb) and BTA14 (from 24.14 Mb to 26.99 Mb). Therefore, we further investigated the broader genomic regions, specifically, 1 Mb upstream and downstream of the genomic region from 23.14 Mb to 27.99 Mb on BTA14, and 40.18 Mb to 42.98 Mb and 37.37 Mb to 39.99 Mb on BTA6 for the CWT.

We identified several positional candidate genes for CWT in BTA6, including *LOC782905*, *SLIT2*, *PACRGL*, *KCNIP4*, *LAP3*, *FAM184B*, and *NCAPG* (Table 5). The *SLIT2* gene was previously detected in Hanwoo cattle, associated with CWT using a population of 427 Hanwoo steers [46]. The *KCNIP4* gene, identified in Canchim beef cattle, has been associated with weaning weight traits and plays a role in calcium ion binding, as well as potassium and voltage-gated ion channel activity [47]. The *LAP3* gene, encoding leucine aminopeptidase, has been associated with milk production and protein concentration in cattle [48] and has been suggested as a candidate gene for improving growth traits in sheep breeding [49]. The *FAM184B* gene, along with four other genes, has been reported in Simmental cattle to be associated with bone weight [50].

In BTA14, we identified 10 positional and functional known candidate genes, namely, *RP1*, *XKR4*, *LYN*, *RPS20*, *MOS*, *FAM110B*, *UBXN2B*, *CYP7A1*, *SDCBP*, *NSMAF*, *TOX*, and *CA8* (Table 5). Additionally, four uncharacterized genomic locations (*LOC107133115*, *LOC101902490*, *LOC101902713*, and *LOC107133116*) were identified. This genomic location has been previously detected by several studies in Hanwoo cattle [12,44,51], suggesting that BTA14 may be a hotspot for genetic variants affecting carcass weight, backfat thickness, and eye muscle area. Similar results have been observed in composite beef cattle breeds, where BTA14 and BTA6 harbored significant loci for genetic variants [24]. 

The *RP1* (retinitis pigmentosa-1) gene, associated with sensory functions causing progressive retinal degeneration, has been observed to have frameshift variants in Normande cattle [52]. Variation in the *XKR4* (XK, Kell blood group complex subunit-related family, member 4) gene has been significantly associated with subcutaneous rump fat thickness in a GWAS of seven breeds of cattle [53]. The *FAM110B* (family with sequence similarity 110) gene plays a specific role in cell cycle progression in yeast cells [54]. The *CYP7A1* (cholesterol 7α-hydroxylase) gene has a key regulatory function in bile acid synthesis, which is important for the regulation of lipid, glucose, and energy homeostasis [55]. The *TOX* (thymocyte selection-associated HMG-box) gene, which plays a critical role in brain development in mouse models, has been associated with reproductive traits in Nelore cattle [56,57]. Mutations in the *CA8* (carbonic anhydrase related protein 8) gene, which is related to a genetic disease in humans, can cause a novel syndrome characterized by ataxia and mild mental retardation, with a predisposition to quadrupedal gait [58]. The *CHD7* gene, previously identified in Hanwoo on BTA14, has been associated with carcass weight at the 5% chromosome-wise level [44].

To expand the search for possible candidate genes associated with carcass weight, we extended the physical location by 1 Mb upstream and downstream of our candidate region. This search led us to identify additional genes, such as *TGS1*, *TMEM68*, *SOX17*, *MRPL15*, *LYPLA1*, *TCEA1*, *RGS20*, *ATP6V1H*, *OPRK1*, *NPBWR1*, *RB1CC1*, and *RAB2A*, located on BTA14 within 4.85 Mb intervals. Well-known genes, including *PLAG1*, *CHCHD7*, *SDR16C5*, *SDR16C6*, *PENK*, and *IMPAD1*, previously reported in many studies with several beef cattle breeds, may also have associations with carcass weight [9,44,59].

The most significant candidate genes, *PLAG1* and *CHCHD7*, have been associated with carcass weight and stature in several cattle breeds, including Hanwoo [60,61,62,63]. The *TMEM68* gene, identified in Nelore cattle, has 64 most significant SNPs associated with reproduction traits [64]. The *LYPLA1* (lysophospholipase 1) gene, previously involved in the regulation of appetite in rat stomach, and the *TMEM68* (transmembrane protein 68) gene, expressed in bovine rumen, abomasum, intestine, and adipose tissue, may affect lipid biosynthesis and contribute to variation in average daily gain (ADG), average daily feed intake (ADFI), and residual feed intake (RFI) in crossbred steers [65]. 

The genes *LYN*, *RPS20*, *MOS*, *PLAG1*, and *CHCHD7*, located on BTA14 (24.87 Mb ~ 25.10 Mb), have been identified as responsible for 1.89% and 2.38% variance in backfat thickness and rump fat thickness in Nelore cattle [66]. Additionally, Fink et al. [62] found that these candidate genes, along with *SDR16C5*, *SDR16C6*, *PENK*, and *IMPAD1*, located in the same genomic region, had significant pleiotropic effects on body weight and milk characteristics in New Zealand Holstein-Friesians, Jerseys, and their crosses. The *NCAPG* (non-SMC condensin 1 complex subunit G) and *LCORL* (ligand dependent nuclear receptor corepressor-like) genes are located on BTA6 between 38.78 to 38.93 Mb, based on the *Bos taurus* UMD 3.1.1 genome assembly. This region aligns with our candidate region captured using the GBLUP method, where GEBVs were used as response variables. Mutations in the *NCAPG* and *LCORL* genes have been associated with carcass weight and body frame size, and they are expressed in the adipose and muscle tissues of beef cattle, potentially influencing average daily feed intake (ADFI) and average daily gain (ADG) phenotypes. Various GWAS have indicated that the *NCAPG* and *LCORL* genes are linked to growth, body size, carcass weight, and carcass composition phenotypes [65]. Moreover, the involvement of the *NCAPG* and *LCORL* gene loci in carcass weight and carcass fat has also been confirmed in Angus, Charolais, and Limousine cattle breeds [63]. 

For the eye muscle area trait, two chromosomal regions on BTA6 were identified. The first region, spanning from 38.37 Mb to 38.99 Mb with 0.62 Mb intervals, harbors five genes (*IBSP*, *LAP3*, *FAM184B*, *NCAPG*, and *LCORL*) (Table 5). The second region, ranging from 61.61 Mb to 63.61 Mb with 2 Mb intervals, harbors two genes (*SLC30A9* and *BEND4*), along with an additional eight genes (*APBB2*, *UCHL1*, *LIMCH1*, *PHOX2B*, *TMEM33*, *SHISA3*, *ATP8A1*, and *GRXCR1*). In the mouse model, the *IBSP* (integrin binding sialoprotein) gene, a major structural component of the bone matrix, has been implicated in bone diseases and skeletal development [67]. The *ATP8A1* gene has been suggested as a strong candidate gene for feed conversion efficiency (FCE) traits in the Sanga cattle population [68]. Furthermore, 14 additional genes, including *FAM13A*, *HERC3*, *NAP1L5*, *PYURF*, *PIGY*, *HERC5*, *HERC6*, *PPM1K*, *ABCG2*, *PKD2*, *SPP1*, *MEPE*, *MED28*, and *DCAF16*, are located within 1 Mb distance from the position on BTA6 (38.37~38.99 Mb). A significant QTL region harboring some of these genes (*HERC3*, *HERC5*, *HERC6*, *IBSP*, and *SPP1*) was reported in the Australian sheep population, associated with gastrointestinal parasitic resistance [69]. The *ABCG2* (ATP binding cassette subfamily G member 2) gene has been identified as a potential candidate gene for facial eczema in sheep [70] and is also involved in multidrug resistance [71]. The *SPP1* gene encodes a protein involved in the toll-like receptors (TLR) signaling pathway. In the chromosomal region on BTA14, ranging from 23.14 to 27.99 Mb (2.85 Mb intervals), we identified 20 potential candidate genes for CWT, which were also associated with EMA in our study. This association suggests a possible pleiotropic relationship between the two traits. 

For backfat thickness, three genes were identified in the chromosomal region on BTA14, ranging from 31.01 to 31.98 Mb (0.97 Mb intervals): *CYP7B1*, *ARMC1*, and *PDE7A* (Table 5). The *CYP7B1* gene, which encodes a steroid cytochrome P450 7α-hydroxylase, has been found to be associated with hereditary spastic paraplegia type 5A, a neurodegenerative disorder in humans [72]. Within a 1 Mb window around the SNP on BTA14 (30.01 ~ 32.98 Mb), an additional 13 annotated genes were found, which may be associated with BF. These genes include *MIR124A-2*, *BHLHE22*, *MTFR1*, *DNAJC5B*, *TRIM55*, *CRH*, *ZSCAN5B*, *RRS1*, *ADHFE1*, *C14H8orf46*, *MYBL1*, *VCPIP*, and *SGK3*, spanning a genomic location of 2.97 Mb. The *CRH* gene, which encodes corticotropin releasing hormone, has been suggested as a potential candidate gene for marbling and the accumulation of subcutaneous fat depth in Wagyu x Limousine crossbreed populations [73]. It has been found to increase locomotor activity and stimulate the hypothalamic–pituitary–adrenal (HPA) axis, which is associated with abdominal fat deposition [74]. The *SGK3* (serum and glucocorticoid-inducible kinase 3) gene plays an important role in bone mineralization and renal tubular phosphate excretion in mouse models [75].

For the marbling score, we identified one SNP (rs110025998) on BTA13 at the 58.14 Mb physical position. This SNP is located within two intronic variants, *LOC783163* and *C13H20orf85*. To explore further, we expanded our search window by approximately 1 Mb in both directions and discovered 18 nearby positional candidate genes, including *SYCP2*, *PHACTR3*, *EDN3*, *ZNF831*, *PRELID3B*, *ATP5E*, *TUBB1*, *CTSZ*, *NELFCD*, *GNAS*, *MIR6123*, *NPEPL1*, *STX16*, *APCDD1L*, *VAPB*, *RAB22A*, and *PMEPA1*. Among these genes, *CTSZ*, *GNAS*, and *RAB22* have been studied (Table 5) in pig populations and are associated with meat color traits [76]. The *VAPB* (Vesicle associated membrane protein associated protein B) gene encodes the VAP protein family, which plays diverse roles in regulating neurotransmitter release, vesicle trafficking, lipid binding and transfer proteins, maintaining ER/golgi architecture, and the unfolded protein response [77]. It has also been found to be overexpressed in human breast cancer. Additionally, *RAB22A*, a member of the proto-oncogene RAS family, plays an important role in the formation, trafficking, and metabolism of exosomes. It is associated with the occurrence and development of multiple human cancers and lymph node metastasis [78]. However, the specific roles of these nearby candidate genes in relation to marbling traits in cattle remain unclear, and further studies are needed to elucidate their significance.

### 3.5. Functional Classification of Annotated Candidate Gene

We analyzed a total of 107 candidate genes within and nearby regions associated with gene ontology (GO) terms related to selected molecular functions, biological processes, and cellular components. The GO analysis revealed that out of the 107 genes, 91 were involved in biological process, 76 genes were associated with cellular components, and 76 genes had molecular functions (Supplementary Material Figure S9). To gain a better understanding of the functional implications of these genes, we also conducted an analysis of biological pathways and protein classes encoded by the genes. 

The PANTHER pathway analysis identified 19 genes that were part of specific pathways (Supplementary Material Figure S10). Some of these pathways, such as the Cadherin signaling pathway, Huntington disease, Parkinson disease, and gonadotropin-releasing hormone receptor pathway, had been previously identified in Hanwoo [79] and were also confirmed in our study. Additionally, some pathways (P00026, P00027, and P00049) involved two genes each. Among the 107 genes, 64 were classified into 17 protein coding classes that are necessary for the functioning of living systems in cattle. Proteins belonging to major classes, such as hydrolase, nucleic acid binding, enzyme modulator, and transporter, were particularly noteworthy. 

Further details regarding the GO terms and KEGG pathways associated with the identified genes, as obtained through DAVID bioinformatics resources using *Bos taurus* as the background, are provided in Supplementary Material Table S4. The candidate genes for the four carcass traits in Hanwoo were annotated and clustered into seven distinct groups. The largest number of annotated genes, such as 6, 4, 6, 11, 13, 7, and 25, were clustered together from 1~7 cluster.

## 4. Conclusions

In conclusion, our study successfully identified significant genetic regions and candidate genes associated with important beef carcass traits in Hanwoo cattle, such as carcass weight, eye muscle area, backfat thickness, and marbling score. Through genome-wide association analysis using a large cattle population, we detected 129 genome-wide significant SNPs, with BTA6, BTA13, and BTA14 being the most prominent genomic regions. We also found a shared genomic region on BTA14 associated with carcass weight and eye muscle area, indicating a genetic relationship between these traits. Our findings emphasize the complex genetic architecture of Hanwoo carcass quality traits, influenced by multiple SNPs in various genes. Further research is needed to validate and confirm these results, providing a deeper understanding of the genetic basis of Hanwoo carcass traits and paving the way for improved breeding and selection strategies in the future.

## Figures and Tables

**Table 1:** Summary statistics for carcass traits in Hanwoo population.

Individuals	Traits	Mean	SD	Min	Max	CV (%)
All (9302)	CWT (kg)	430.46	61.08	159	692	14.19
	EMA (cm^2^)	94.16	13.24	34	156	14.06
	BF (mm)	14.09	5.13	2	47	36.40
	MS (score 1–9)	5.83	2.09	1	9	35.81
Steers (7328)	CWT (kg)	445.17	48.93	180	692	10.99
	EMA (cm^2^)	96.70	11.98	34	156	12.39
	BF (mm)	14.53	4.98	2	47	34.27
	MS (score 1–9)	6.31	1.78	1	9	28.21

SD, standard deviation; Min, minimum; Max, maximum; CV, coefficient of variation; CWT, carcass weight; EMA, eye muscle area; BF, backfat thickness; MS, marbling score.

**Table 2:** Estimates of additive genetic variance, residual variance, phenotypic variance, and heritability for four carcass traits in Hanwoo.

Method	Traits	$\sigma_{a}^{2}$	$\sigma_{e}^{2}$	$\sigma_{P}^{2}$	$h^{2}$
GBLUP	CWT	674.69	931.26	1606.00	0.42
	EMA	42.13	73.71	115.84	0.36
	BF	7.73	13.89	21.62	0.36
	MS	1.18	1.35	2.53	0.47
Bayes B	CWT	817.52	1742.63	925.11	0.47
	EMA	43.15	117.75	74.60	0.37
	BF	7.94	21.84	13.89	0.36
	MS	1.04	2.47	1.43	0.42

$\sigma_{a}^{2}$, genetic variance; $\sigma_{e}^{2}$, residual variance; $\sigma_{p}^{2}$, phenotypic variance; $h^{2}$, heritability; GBLUP, genomic best linear unbiased prediction.

**Table 3:** Genome-wide significant common SNPs (top ten for each trait) identified by GBLUP and Bayes B method, underlying CWT, EMA, BF, and MS traits in Hanwoo using de-regressed genomic estimated breeding values.

SNP	BTA:Position (bp)	Estimate ± SE	−log_10_P	Nearest Gene	Consequence Type
Carcass weight					
rs384536472	14:24497726	3.500 ± 0.088	12.787	-	-
rs384017132	14:24524210	3.505 ± 0.088	12.774	*XKR4*	IV
rs721927483	14:24973953	3.517 ± 0.088	12.774	*MOS*	DGV
rs134315607	14:26311106	3.520 ± 0.089	12.669	*UBXN2B*	DGV
rs42646659	14:26443481	3.604 ± 0.089	12.732	*NSMAF*	DGV
rs41725159	14:26619895	3.578 ± 0.088	12.810	*LOC107133116*	DGV
rs41725162	14:26621673	3.578 ± 0.088	12.763	*LOC107133116*	DGV
rs42406058	14:26848418	3.592 ± 0.088	12.666	*TOX*	IV
rs42406039	14:26859737	3.595 ± 0.088	12.666	*TOX*	IV
rs42404941	14:26967218	3.108 ± 0.078	12.686	*TOX*, *CA8*	IntGV
Eye muscle area					
rs467005433	6:38530564	4.405 ± 0.137	8.295	-	-
rs798880362	6:38580679	4.413 ± 0.137	8.323	*LAP3*	IV
rs432843769	6:38581067	4.052 ± 0.133	7.521	*LAP3*	IV
rs110834363	6:38939012	3.351 ± 0.111	7.308	*LCORL*	IV
rs210258477	14:24497726	0.503 ± 0.017	7.220	-	-
rs42646659	14:24524210	0.504 ± 0.017	7.218	*XKR4*	IV
rs209439851	14:26443481	0.507 ± 0.018	7.137	*NSMAF*	DGV
rs41724548	14:26743126	0.505 ± 0.017	7.117	*TOX*	IV
rs41724547	14:26746062	0.505 ± 0.017	7.119	*TOX*	IV
rs41724028	14:26776546	0.505 ± 0.017	7.127	*TOX*	IV
Backfat thickness					
rs109694900	14:31028004	0.400 ± 0.015	5.693	*CYP7B1*	IV
rs110883161	14:31058827	0.406 ± 0.015	5.776	*CYP7B1*	IV
rs109108816	14:31086712	0.415 ± 0.015	5.965	*CYP7B1*	IV
rs109546980	14:31219729	0.569 ± 0.019	7.457	*LOC104974032*, *ARMC1*	IntGV
rs42743206	14:31244368	0.556 ± 0.019	7.179	*LOC104974032*, *ARMC1*	IntGV
rs137211394	14:31264561	0.534 ± 0.018	6.808	*LOC104974032*, *ARMC1*	IntGV
rs41639002	14:31322421	0.556 ± 0.019	7.186	*LOC104974032*, *ARMC1*	IntGV
rs110366862	14:31362273	0.557 ± 0.019	7.211	*LOC104974032*, *ARMC1*	IntGV
rs41619147	14:31377493	0.568 ± 0.019	7.428	*LOC104974032*, *ARMC1*	IntGV
rs137843592	14:31944251	2.297 ± 0.085	5.980	*PDE7A*, *LOC100299601*	IntGV
Marbling score					
rs110025998	13:58140449	−0.055 ± 0.003	4.064	*LOC783163*, *C13H20orf85*	IV

SNP, single nucleotide polymorphism; BTA, *Bos taurus* autosome; bp, base pair (kb); SE, standard error; IntGV, intergenic variant; IV, intron variant; DGV, downstream gene variant.

**Table 4:** Genome-wide significant common SNPs (top ten for each trait) identified by GBLUP and Bayes B method, underlying CWT, EMA, BF, and MS traits in Hanwoo using genomic estimated breeding values.

SNP	BTA:Position (bp)	Estimate ± SE	−log_10_P	Nearest Gene	Consequence Type
Carcass weight					
rs110092040	14:24973953	18.620 ± 0.449	13.850	MOS	DGV
rs42304778	14:26162492	19.120 ± 0.453	13.833	*FAM110B*, *LOC101902490*	IntGV
rs42304792	14:26173037	19.110 ± 0.453	13.832	*FAM110B*, *LOC101902490*	IntGV
rs42304759	14:26196375	18.180 ± 0.449	13.829	*LOC101902490*, *UBXN2B*	IntGV
rs110634307	14:26223753	18.610 ± 0.451	13.821	*LOC101902490*, *UBXN2B*	IntGV
rs42303720	14:26264142	18.620 ± 0.450	13.819	*LOC101902490*, *UBXN2B*	IntGV
rs43083563	14:26311106	18.680 ± 0.451	13.818	*UBXN2B*	DGV
rs41614868	14:26351959	18.000 ± 0.443	13.812	*TRNAG-CCC*	DGV
rs209439851	14:26443481	19.130 ± 0.450	13.756	*NSMAF*	DGV
rs41725705	14:26570145	19.010 ± 0.449	13.727	*NSMAF*, *LOC107133116*	IntGV
Eye muscle area					
rs210258477	14:24497726	3.100 ± 0.102	7.710	-	-
rs42646659	14:24524210	3.107 ± 0.102	7.704	*XKR4*	IV
rs42304778	14:26162492	3.120 ± 0.104	7.538	*FAM110B*, *LOC101902490*	IntGV
rs42304792	14:26173037	3.125 ± 0.104	7.542	*FAM110B*, *LOC101902490*	IntGV
rs209439851	14:26443481	3.122 ± 0.103	7.615	*NSMAF*	DGV
rs41725705	14:26570145	3.103 ± 0.103	7.545	*NSMAF*, *LOC107133116*	IntGV
rs41725159	14:26619895	3.097 ± 0.103	7.533	*LOC107133116*	DGV
rs41724548	14:26743126	3.110 ± 0.103	7.577	*TOX*	IV
rs41724547	14:26746062	3.109 ± 0.103	7.577	*TOX*	IV
rs41724028	14:26776546	3.111 ± 0.103	7.587	*TOX*	IV

SNP, single nucleotide polymorphism; BTA, *Bos taurus* autosome; bp, base pair (kb); SE, standard error; IntGV, intergenic variant; IV, intron variant; DGV, downstream gene variant.

**Table 5:** Candidate genes associated with carcass traits in Hanwoo steers.

Traits	Genes	Name	BTA	QTL Position (Mb)
CWT	*SLIT2*	Slit guidance ligand 2	6	41.20–41.68
CWT	*PACRGL*	Parkin coregulated like	6	41.69–41.71
CWT	*KCNIP4*	Potassium voltage-gated channel interacting protein 4	6	41.71–43.02
CWT	*LAP3*	Leucine aminopeptidase 3	6	38.57–38.60
CWT	*FAM184B*	Family with sequence similarity 184 member B	6	38.61–38.74
CWT	*NCAPG*	Non-SMC condensin I complex subunit G	6	38.71–38.81
CWT	*RP1*	Axonemal microtubule associated	14	23.99–24.00
CWT	*XKR4*	XK related 4	14	24.30–24.61
CWT	*LYN*	LYN proto-oncogene, Src family tyrosine kinase	14	24.85–24.92
CWT	*RPS20*	Ribosomal protein S20	14	24.96–24.96
CWT	*MOS*	MOS proto-oncogene, serine/threonine kinase	14	24.98–24.98
CWT	*FAM110B*	Family with sequence similarity 110 member B	14	26.05–26.12
CWT	*UBXN2B*	UBX domain protein 2B	14	26.27–26.31
CWT	*CYP7A1*	Cytochrome P450 family 7 subfamily A member 1	14	26.35–26.36
CWT	*SDCBP*	Syndecan binding protein	14	26.41–26.45
CWT	*NSMAF*	Neutral sphingomyelinase activation associated factor	14	26.45–26.51
CWT	*TOX*	Thymocyte selection associated high mobility group box	14	26.63–26.94
CWT	*CA8*	Carbonic anhydrase 8	14	27.64–27.72
EMA	*IBSP*	Integrin binding sialoprotein	6	38.31–38.32
EMA	*LAP3*	Leucine aminopeptidase 3	6	38.57–38.60
EMA	*FAM184B*	Family with sequence similarity 184 member B	6	38.61–38.74
EMA	*LCORL*	Ligand dependent nuclear receptor corepressor like	6	38.84–38.99
EMA	*NCAPG*	Non-SMC condensin I complex subunit G	6	38.71–38.81
EMA	*SLC30A9*	Solute carrier family 30 member 9	6	62.51–62.60
EMA	*BEND4*	BEN domain containing 4	6	62.63–62.66
BF	*CYP7B1*	Cytochrome P450 family 7 subfamily B member 1	14	30.98–31.15
BF	*ARMC1*	Armadillo repeat containing 1	14	31.70–31.72
BF	*PDE7A*	Phosphodiesterase 7A	14	31.80–31.91
MS	*CTSZ*	Cathepsin Z	13	57.89–57.90
MS	*GNAS*	GNAS complex locus	13	58.01–58.05
MS	*VAPB*	VAMP associated protein B and C	13	58.46–58.51
MS	*RAB22A*	RAB22A, member RAS oncogene family	13	58.54–58.59

## Data Availability

The dataset is available from the corresponding author on reasonable request.

## Supplementary Materials

The following are available online at https://www.mdpi.com/article/10.3390/ani13172737/s1, Figure S1: Quantile-quantile (Q-Q) plots for carcass weight in Hanwoo cattle using DGEBV (upper panel), GEBV (middle panel), and residual (lower panel) as predictors in the GBLUP method. Figure S2: Quantile-quantile (Q-Q) plots for eye muscle area in Hanwoo cattle using DGEBV (upper panel), GEBV (middle panel), and residual (lower panel) as predictors in the GBLUP method. Figure S3: Quantile-quantile (Q-Q) plots for backfat thickness in Hanwoo cattle using DGEBV (upper panel), GEBV (middle panel), and residual (lower panel) as predictors in the GBLUP method. Figure S4: Quantile-quantile (Q-Q) plots for marbling score in Hanwoo cattle using DGEBV (upper panel), GEBV (middle panel), and residual (lower panel) as predictors in the GBLUP method. Figure S5: Genome-wide proportion of variance for carcass weight in Hanwoo cattle using DGEBV (upper panel), GEBV (middle panel), and residual (lower panel) as predictors in the Bayes B method. Figure S6: Genome-wide proportion of variance for eye muscle area in Hanwoo cattle using DGEBV (upper panel), GEBV (middle panel), and residual (lower panel) as predictors in the Bayes B method. Figure S7: Genome-wide proportion of variance for backfat thickness in Hanwoo cattle using DGEBV (upper panel), GEBV (middle panel), and residual (lower panel) as predictors in the Bayes B method. Figure S8: Genome-wide proportion of variance for marbling score in Hanwoo cattle using DGEBV (upper panel), GEBV (middle panel), and residual (lower panel) as predictors in the Bayes B method. Figure S9: The PANTHER GO (Biological process, cellular components, and molecular function) is based on 107 selected genes from GWAS study, showing the percentage of gene hit against total process hits. Figure S10: The PANTHER pathway (top) and protein class (down) based on 107 selected genes from GWAS study, showing the percent of gene hit against total process hits. Table S1: Estimates of genetic variance, residual variance, phenotypic variance, and heritability for four carcass traits in Hanwoo using 5-fold cross validation. Table S2. Genome-wide significant common SNPs identified by GBLUP and Bayes B method, underlying CWT, EMA, BF, and MS traits in Hanwoo using de-regressed genomic estimated breeding values. Table S3. Genome-wide significant common SNPs identified by GBLUP and Bayes B method, underlying CWT, EMA, BF, and MS traits in Hanwoo using genomic estimated breeding values. Table S4: Functional cluster enrichment analysis of gene via DAVID database for the carcass traits in Hanwoo.

## References

[1] Bovine Genome S., Analysis C., Elsik C.G., Tellam R.L., Worley K.C., Gibbs R.A., Muzny D.M., Weinstock G.M., Adelson D.L., Eichler E.E. (2009). The genome sequence of taurine cattle: A window to ruminant biology and evolution. Science.

[2] Mei C., Wang H., Zhu W., Wang H., Cheng G., Qu K., Guang X., Li A., Zhao C., Yang W. (2016). Whole-genome sequencing of the endangered bovine species Gayal (*Bos frontalis*) provides new insights into its genetic features. Sci. Rep..

[3] Matukumalli L.K., Lawley C.T., Schnabel R.D., Taylor J.F., Allan M.F., Heaton M.P., O’Connell J., Moore S.S., Smith T.P., Sonstegard T.S. (2009). Development and characterization of a high density SNP genotyping assay for cattle. PLoS ONE.

[4] Hayes B.J., Bowman P.J., Chamberlain A.C., Verbyla K., Goddard M.E. (2009). Accuracy of genomic breeding values in multi-breed dairy cattle populations. Genet. Sel. Evol..

[5] Kim S., Alam M., Park M.N. (2017). Breeding initiatives for Hanwoo cattle to thrive as a beef industry—A review study. J. Anim. Breed. Genom..

[6] Chung K.Y., Lee S.H., Cho S.H., Kwon E.G., Lee J.H. (2018). Current situation and future prospects for beef production in South Korea—A review. Asian-Australas. J. Anim. Sci..

[7] Hindorff L.A., Sethupathy P., Junkins H.A., Ramos E.M., Mehta J.P., Collins F.S., Manolio T.A. (2009). Potential etiologic and functional implications of genome-wide association loci for human diseases and traits. Proc. Natl. Acad. Sci. USA.

[8] Brodie A., Azaria J.R., Ofran Y. (2016). How far from the SNP may the causative genes be?. Nucleic Acids Res..

[9] Lee S.H., Choi B.H., Lim D., Gondro C., Cho Y.M., Dang C.G., Sharma A., Jang G.W., Lee K.T., Yoon D. (2013). Genome-wide association study identifies major loci for carcass weight on BTA14 in Hanwoo (*Korean cattle*). PLoS ONE.

[10] Sharma A., Dang C.G., Kim K.S., Kim J.J., Lee H.K., Kim H.C., Yeon S.H., Kang H.S., Lee S.H. (2014). Validation of genetic polymorphisms on BTA14 associated with carcass trait in a commercial Hanwoo population. Anim. Genet..

[11] Bedhane M., van der Werf J., Gondro C., Duijvesteijn N., Lim D., Park B., Park M.N., Hee R.S., Clark S. (2019). Genome-Wide Association Study of Meat Quality Traits in Hanwoo Beef Cattle Using Imputed Whole-Genome Sequence Data. Front. Genet..

[12] Bhuiyan M.S.A., Lim D., Park M., Lee S., Kim Y., Gondro C., Park B., Lee S. (2018). Functional Partitioning of Genomic Variance and Genome-Wide Association Study for Carcass Traits in Korean Hanwoo Cattle Using Imputed Sequence Level SNP Data. Front. Genet..

[13] Edea Z., Jeoung Y.H., Shin S.S., Ku J., Seo S., Kim I.H., Kim S.W., Kim K.S. (2018). Genome-wide association study of carcass weight in commercial Hanwoo cattle. Asian-Australas. J. Anim. Sci..

[14] Kim H.J., de Las Heras-Saldana S., Moghaddar N., Lee S.H., Lim D., van der Werf J.H.J. (2022). Genome-wide association study for carcass traits in Hanwoo cattle using additional relatives’ information of non-genotyped animals. Anim. Genet..

[15] Purcell S., Neale B., Todd-Brown K., Thomas L., Ferreira M.A., Bender D., Maller J., Sklar P., de Bakker P.I., Daly M.J. (2007). PLINK: A tool set for whole-genome association and population-based linkage analyses. Am. J. Hum. Genet..

[16] Browning B.L., Tian X., Zhou Y., Browning S.R. (2021). Fast two-stage phasing of large-scale sequence data. Am. J. Hum. Genet..

[17] Gilmour A.R., Gogel B.J., Cullis B.R., Welham S.J., Thompson R. (2021). ASReml User Guide Release 4.2 Functional Specification.

[18] VanRaden P.M. (2008). Efficient methods to compute genomic predictions. J. Dairy Sci..

[19] Yang J., Lee S.H., Goddard M.E., Visscher P.M. (2011). GCTA: A tool for genome-wide complex trait analysis. Am. J. Hum. Genet..

[20] Garrick D.J., Taylor J.F., Fernando R.L. (2009). Deregressing estimated breeding values and weighting information for genomic regression analyses. Genet. Sel. Evol..

[21] Fernando R.L., Garrick D.J. (2008). GenSel-User Manual for a Portfolio of Genomic Selection Related Analyses.

[22] Meuwissen T.H., Hayes B.J., Goddard M.E. (2001). Prediction of total genetic value using genome-wide dense marker maps. Genetics.

[23] Habier D., Fernando R.L., Kizilkaya K., Garrick D.J. (2011). Extension of the bayesian alphabet for genomic selection. BMC Bioinform..

[24] Hay E., Roberts A. (2018). Genome-wide association study for carcass traits in a composite beef cattle breed. Livest. Sci..

[25] Mi H., Ebert D., Muruganujan A., Mills C., Albou L.-P., Mushayamaha T., Thomas P.D. (2020). PANTHER version 16: A revised family classification, tree-based classification tool, enhancer regions and extensive API. Nucleic Acids Res..

[26] Huang D.W., Sherman B.T., Lempicki R.A. (2009). Systematic and integrative analysis of large gene lists using DAVID bioinformatics resources. Nat. Protoc..

[27] Turner S.D. (2014). qqman: An R package for visualizing GWAS results using Q-Q and manhattan plots. bioRxiv.

[28] Kwon A., Srikanth K., Lee E., Kim S., Chung H. (2016). Confirmation of genotypic effects for the bovine APM1 gene on marbling in Hanwoo cattle. J. Anim. Sci. Technol..

[29] Haque M.A., Iqbal A., Bae H., Lee S.E., Park S., Lee Y.M., Kim J.J. (2023). Assessment of genomic breeding values and their accuracies for carcass traits in Jeju Black cattle using whole-genome SNP chip panels. J. Anim. Breed. Genet..

[30] Mehrban H., Lee D.H., Moradi M.H., IlCho C., Naserkheil M., Ibanez-Escriche N. (2017). Predictive performance of genomic selection methods for carcass traits in Hanwoo beef cattle: Impacts of the genetic architecture. Genet. Sel. Evol..

[31] Park B., Choi T., Kim S., Oh S.H. (2013). National genetic evaluation (system) of hanwoo (korean native cattle). Asian-Australas. J. Anim. Sci..

[32] Do C., Park B., Kim S., Choi T., Yang B., Park S., Song H. (2016). Genetic Parameter Estimates of Carcass Traits under National Scale Breeding Scheme for Beef Cattle. Asian-Australas. J. Anim. Sci..

[33] Utrera A.R., Van Vleck L.D. (2004). Heritability estimates for carcass traits of cattle: A review. Genet. Mol. Res. GMR.

[34] Kim J., Kim D., Lee J., Lee C. (2010). Genetic Relationship between Carcass Traits and Carcass Price of Korean Cattle. Asian-Australas. J. Anim. Sci..

[35] Koh D., Lee J., Won S., Lee C., Kim J. (2014). Genetic relationships of carcass traits with retail cut productivity of hanwoo cattle. Asian-Australas. J. Anim. Sci..

[36] Oyama K. (2011). Genetic variability of Wagyu cattle estimated by statistical approaches. Anim. Sci. J.=Nihon Chikusan Gakkaiho.

[37] Munim T., Oikawa T., Ibi T., Kunieda T. (2013). Genetic relationship of body measurement traits at early age with carcass traits in Japanese black cattle. Anim. Sci. J.=Nihon Chikusan Gakkaiho.

[38] MacNeil M.D., Northcutt S.L. (2008). National cattle evaluation system for combined analysis of carcass characteristics and indicator traits recorded by using ultrasound in Angus cattle1. J. Anim. Sci..

[39] Rolf M.M., Garrick D.J., Fountain T., Ramey H.R., Weaber R.L., Decker J.E., Pollak E.J., Schnabel R.D., Taylor J.F. (2015). Comparison of Bayesian models to estimate direct genomic values in multi-breed commercial beef cattle. Genet. Sel. Evol..

[40] Pariacote F., Van Vleck L.D., Hunsley R.E. (1998). Genetic and phenotypic parameters for carcass traits of American Shorthorn beef cattle. J. Anim. Sci..

[41] Moser G., Khatkar M.S., Hayes B.J., Raadsma H.W. (2010). Accuracy of direct genomic values in Holstein bulls and cows using subsets of SNP markers. Genet. Sel. Evol..

[42] Muir W.M. (2007). Comparison of genomic and traditional BLUP-estimated breeding value accuracy and selection response under alternative trait and genomic parameters. J. Anim. Breed. Genet..

[43] Yin T., Pimentel E.C., Konig V.B.U., Konig S. (2014). Strategy for the simulation and analysis of longitudinal phenotypic and genomic data in the context of a temperature × humidity-dependent covariate. J. Dairy Sci..

[44] Li Y., Gao Y., Kim Y.S., Iqbal A., Kim J.J. (2017). A whole genome association study to detect additive and dominant single nucleotide polymorphisms for growth and carcass traits in Korean native cattle, Hanwoo. Asian-Australas. J. Anim. Sci..

[45] Li Y., Kim J.J. (2015). Effective Population Size and Signatures of Selection Using Bovine 50K SNP Chips in Korean Native Cattle (Hanwoo). Evol. Bioinform. Online.

[46] Lee J.H., Li Y., Kim J.J. (2012). Detection of QTL for Carcass Quality on Chromosome 6 by Exploiting Linkage and Linkage Disequilibrium in Hanwoo. Asian-Australas. J. Anim. Sci..

[47] Buzanskas M.E., Grossi D.A., Ventura R.V., Schenkel F.S., Sargolzaei M., Meirelles S.L., Mokry F.B., Higa R.H., Mudadu M.A., da Silva M.V. (2014). Genome-wide association for growth traits in Canchim beef cattle. PLoS ONE.

[48] Cohen-Zinder M., Seroussi E., Larkin D.M., Loor J.J., Everts-van der Wind A., Lee J.H., Drackley J.K., Band M.R., Hernandez A.G., Shani M. (2005). Identification of a missense mutation in the bovine ABCG2 gene with a major effect on the QTL on chromosome 6 affecting milk yield and composition in Holstein cattle. Genome Res..

[49] La Y., Zhang X., Li F., Zhang D., Li C., Mo F., Wang W. (2019). Molecular Characterization and Expression of SPP1, LAP3 and LCORL and Their Association with Growth Traits in Sheep. Genes.

[50] Xia J., Fan H., Chang T., Xu L., Zhang W., Song Y., Zhu B., Zhang L., Gao X., Chen Y. (2017). Searching for new loci and candidate genes for economically important traits through gene-based association analysis of Simmental cattle. Sci. Rep..

[51] Lee K.T., Chung W.H., Lee S.Y., Choi J.W., Kim J., Lim D., Lee S., Jang G.W., Kim B., Choy Y.H. (2013). Whole-genome resequencing of Hanwoo (*Korean cattle*) and insight into regions of homozygosity. BMC Genom..

[52] Michot P., Chahory S., Marete A., Grohs C., Dagios D., Donzel E., Aboukadiri A., Deloche M.C., Allais-Bonnet A., Chambrial M. (2016). A reverse genetic approach identifies an ancestral frameshift mutation in RP1 causing recessive progressive retinal degeneration in European cattle breeds. Genet. Sel. Evol..

[53] Porto Neto L.R., Bunch R.J., Harrison B.E., Barendse W. (2012). Variation in the XKR4 gene was significantly associated with subcutaneous rump fat thickness in indicine and composite cattle. Anim. Genet..

[54] Hauge H., Patzke S., Aasheim H.C. (2007). Characterization of the FAM110 gene family. Genomics.

[55] Li Y., Willer C.J., Ding J., Scheet P., Abecasis G.R. (2010). MaCH: Using sequence and genotype data to estimate haplotypes and unobserved genotypes. Genet. Epidemiol..

[56] Artegiani B., de Jesus Domingues A.M., Bragado Alonso S., Brandl E., Massalini S., Dahl A., Calegari F. (2015). Tox: A multifunctional transcription factor and novel regulator of mammalian corticogenesis. EMBO J..

[57] de Camargo G.M., Costa R.B., de Albuquerque L.G., Regitano L.C., Baldi F., Tonhati H. (2015). Polymorphisms in TOX and NCOA2 genes and their associations with reproductive traits in cattle. Reprod. Fertil. Dev..

[58] Turkmen S., Guo G., Garshasbi M., Hoffmann K., Alshalah A.J., Mischung C., Kuss A., Humphrey N., Mundlos S., Robinson P.N. (2009). CA8 mutations cause a novel syndrome characterized by ataxia and mild mental retardation with predisposition to quadrupedal gait. PLoS Genet..

[59] Magalhaes A.F., de Camargo G.M., Fernandes G.A.J., Gordo D.G., Tonussi R.L., Costa R.B., Espigolan R., Silva R.M., Bresolin T., de Andrade W.B. (2016). Genome-Wide Association Study of Meat Quality Traits in Nellore Cattle. PLoS ONE.

[60] Nishimura S., Watanabe T., Mizoshita K., Tatsuda K., Fujita T., Watanabe N., Sugimoto Y., Takasuga A. (2012). Genome-wide association study identified three major QTL for carcass weight including the PLAG1-CHCHD7 QTN for stature in Japanese Black cattle. BMC Genet..

[61] Fortes M.R., Kemper K., Sasazaki S., Reverter A., Pryce J.E., Barendse W., Bunch R., McCulloch R., Harrison B., Bolormaa S. (2013). Evidence for pleiotropism and recent selection in the PLAG1 region in Australian Beef cattle. Anim. Genet..

[62] Fink T., Tiplady K., Lopdell T., Johnson T., Snell R.G., Spelman R.J., Davis S.R., Littlejohn M.D. (2017). Functional confirmation of PLAG1 as the candidate causative gene underlying major pleiotropic effects on body weight and milk characteristics. Sci. Rep..

[63] Purfield D.C., Evans R.D., Berry D.P. (2019). Reaffirmation of known major genes and the identification of novel candidate genes associated with carcass-related metrics based on whole genome sequence within a large multi-breed cattle population. BMC Genom..

[64] Melo T.P., Fortes M.R.S., Bresolin T., Mota L.F.M., Albuquerque L.G., Carvalheiro R. (2018). Multitrait meta-analysis identified genomic regions associated with sexual precocity in tropical beef cattle. J. Anim. Sci..

[65] Lindholm-Perry A.K., Kuehn L.A., Oliver W.T., Sexten A.K., Miles J.R., Rempel L.A., Cushman R.A., Freetly H.C. (2013). Adipose and muscle tissue gene expression of two genes (NCAPG and LCORL) located in a chromosomal region associated with cattle feed intake and gain. PLoS ONE.

[66] Medeiros de Oliveira Silva R., Bonvino Stafuzza N., de Oliveira Fragomeni B., Miguel Ferreira de Camargo G., Matos Ceacero T., Noely Dos Santos Goncalves Cyrillo J., Baldi F., Augusti Boligon A., Zerlotti Mercadante M.E., Lino Lourenco D. (2017). Genome-Wide Association Study for Carcass Traits in an Experimental Nelore Cattle Population. PLoS ONE.

[67] Bouleftour W., Boudiffa M., Wade-Gueye N.M., Bouet G., Cardelli M., Laroche N., Vanden-Bossche A., Thomas M., Bonnelye E., Aubin J.E. (2014). Skeletal development of mice lacking bone sialoprotein (BSP)--impairment of long bone growth and progressive establishment of high trabecular bone mass. PLoS ONE.

[68] Taye M., Kim J., Yoon S.H., Lee W., Hanotte O., Dessie T., Kemp S., Mwai O.A., Caetano-Anolles K., Cho S. (2017). Whole genome scan reveals the genetic signature of African Ankole cattle breed and potential for higher quality beef. BMC Genet..

[69] Al Kalaldeh M., Gibson J., Lee S.H., Gondro C., van der Werf J.H.J. (2019). Detection of genomic regions underlying resistance to gastrointestinal parasites in Australian sheep. Genet. Sel. Evol..

[70] Duncan E.J., Dodds K.G., Henry H.M., Thompson M.P., Phua S.H. (2007). Cloning, mapping and association studies of the ovine ABCG2 gene with facial eczema disease in sheep. Anim. Genet..

[71] Nakanishi T., Ross D.D. (2012). Breast cancer resistance protein (BCRP/ABCG2): Its role in multidrug resistance and regulation of its gene expression. Chin. J. Cancer.

[72] Roos P., Svenstrup K., Danielsen E.R., Thomsen C., Nielsen J.E. (2014). CYP7B1: Novel mutations and magnetic resonance spectroscopy abnormalities in hereditary spastic paraplegia type 5A. Acta Neurol. Scand..

[73] Wibowo T.A., Michal J.J., Jiang Z. (2007). Corticotropin releasing hormone is a promising candidate gene for marbling and subcutaneous fat depth in beef cattle. Genome.

[74] Perusse L., Rice T., Chagnon Y.C., Despres J.P., Lemieux S., Roy S., Lacaille M., Ho-Kim M.A., Chagnon M., Province M.A. (2001). A genome-wide scan for abdominal fat assessed by computed tomography in the Quebec Family Study. Diabetes.

[75] Bhandaru M., Kempe D.S., Rotte A., Capuano P., Pathare G., Sopjani M., Alesutan I., Tyan L., Huang D.Y., Siraskar B. (2011). Decreased bone density and increased phosphaturia in gene-targeted mice lacking functional serum- and glucocorticoid-inducible kinase 3. Kidney Int..

[76] Hu Z.L., Ramos A.M., Humphray S.J., Rogers J., Reecy J.M., Rothschild M.F. (2011). Use of Genome Sequence Information for Meat Quality Trait QTL Mining for Causal Genes and Mutations on Pig Chromosome 17. Front. Genet..

[77] Rao M., Song W., Jiang A., Shyr Y., Lev S., Greenstein D., Brantley-Sieders D., Chen J. (2012). VAMP-associated protein B (VAPB) promotes breast tumor growth by modulation of Akt activity. PLoS ONE.

[78] Sun L., He M., Xu N., Xu D.H., Ben-David Y., Yang Z.Y., Li Y.J. (2018). Regulation of RAB22A by mir-193b inhibits breast cancer growth and metastasis mediated by exosomes. Int. J. Oncol..

[79] Lee S.H., Park B.H., Sharma A., Dang C.G., Lee S.S., Choi T.J., Choy Y.H., Kim H.C., Jeon K.J., Kim S.D. (2014). Hanwoo cattle: Origin, domestication, breeding strategies and genomic selection. J. Anim. Sci. Technol..

