# Supplementary material for: Genome-Wide Association Study to Identify QTL for Carcass Traits in Korean Hanwoo Cattle
Source: Animals (Basel). 2023 Aug 28;13(17):2737. doi: 10.3390/ani13172737 (PMC10486602; doi:10.3390/ani13172737)
Supplement: Supplementary file 1 [file animals-13-02737-s001.zip › animals-2540008-supplementary.pdf]

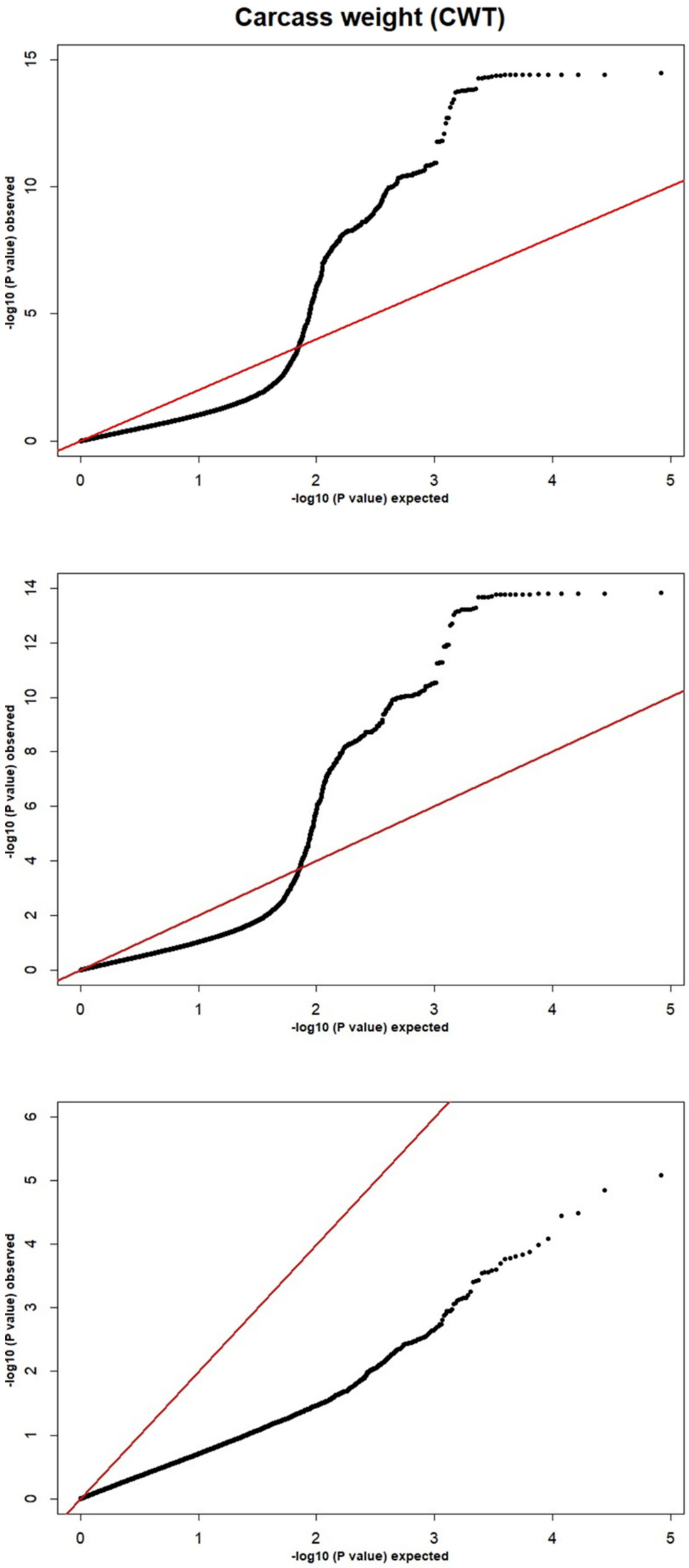

Figure S1. Quantile-quantile (Q-Q) plots for carcass weight in Hanwoo cattle using DGEBV (upper panel), GEBV (middle panel), and residual (lower panel) as predictors in the GBLUP method. Q–Q plot showing the late separation between observed and expected *p*-values (-$\log_{10}$P). The red line represents the 95% concentration band under the null hypothesis of no association.

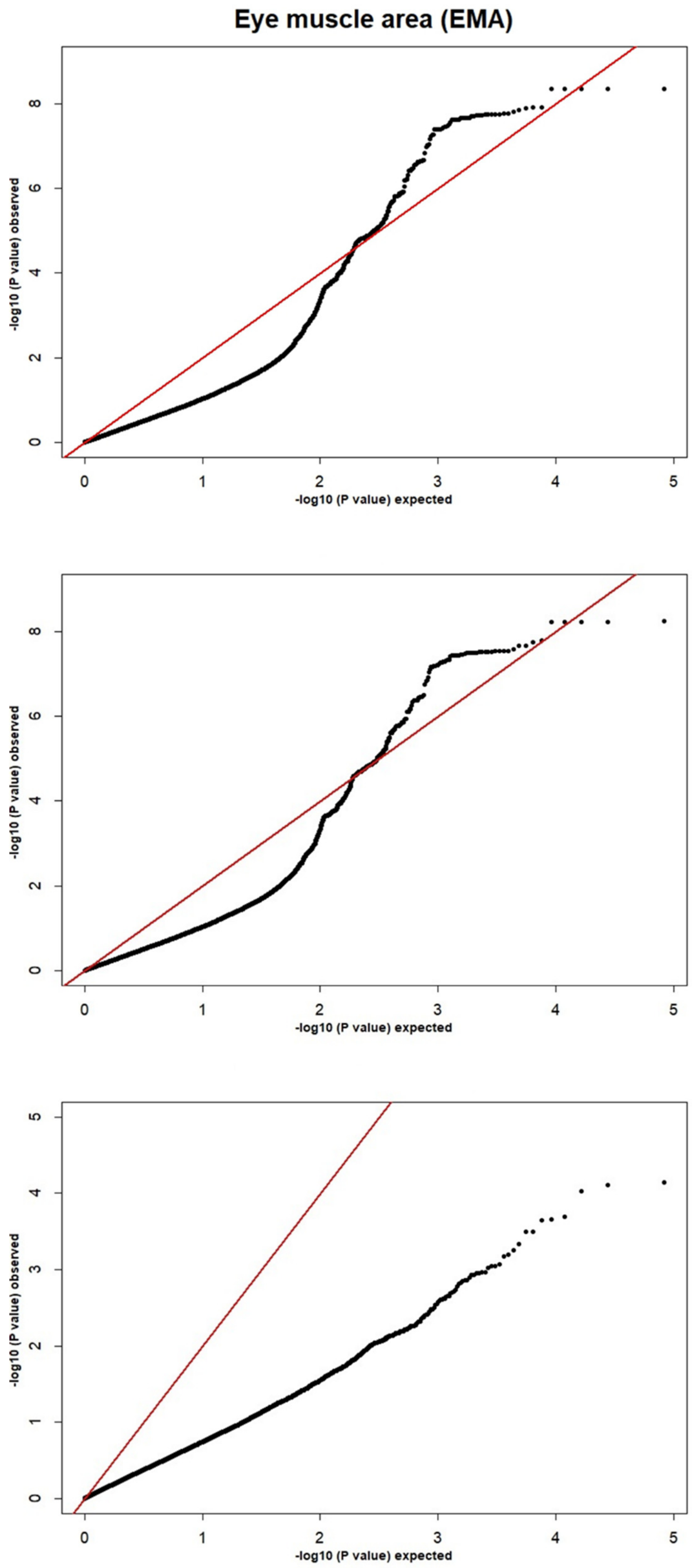

Figure S2. Quantile-quantile (Q-Q) plots for eye muscle area in Hanwoo cattle using DGEBV (upper panel), GEBV (middle panel), and residual (lower panel) as predictors in the GBLUP method. Q–Q plot showing the late separation between observed and expected *p*-values (-log$_{10}$P). The red line represents the 95% concentration band under the null hypothesis of no association.

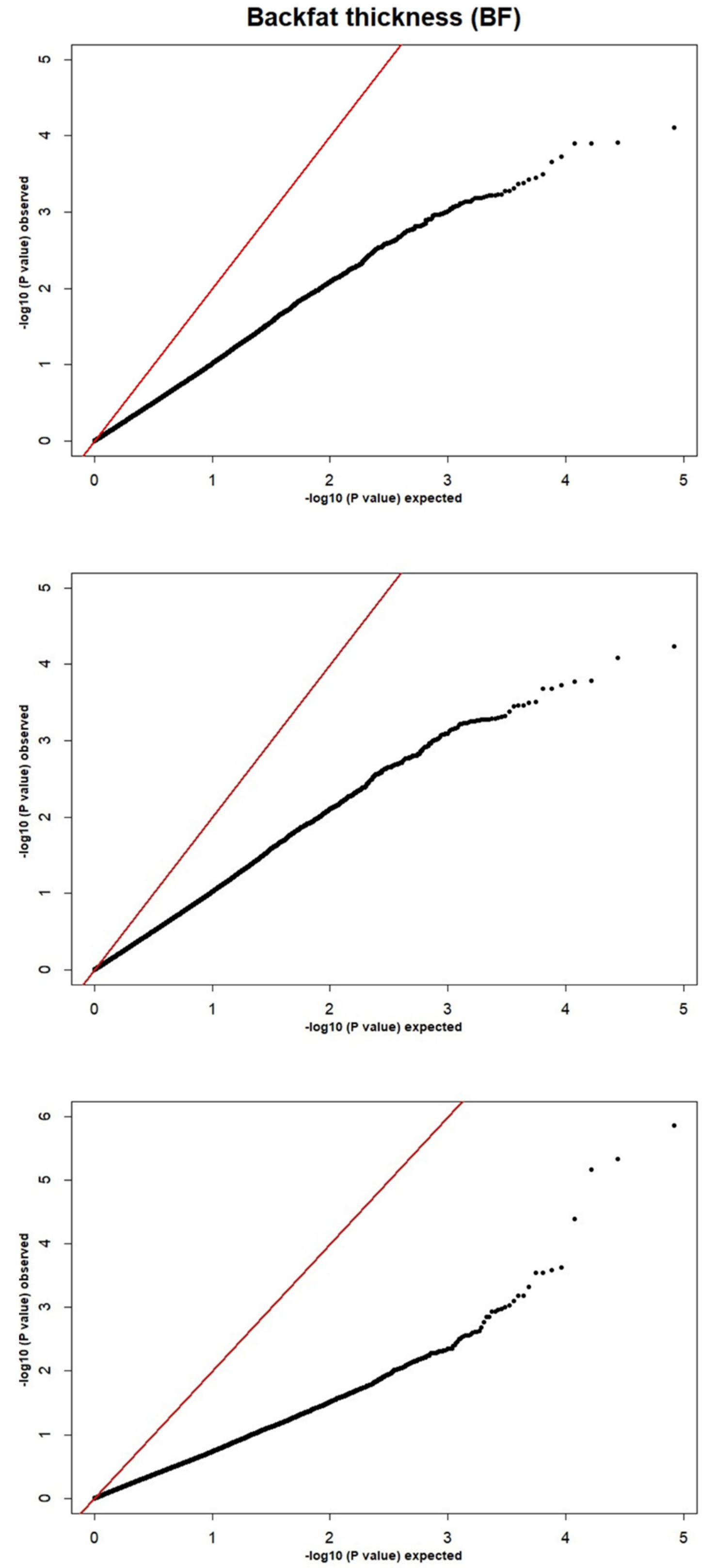

Figure S3. Quantile-quantile (Q-Q) plots for backfat thickness in Hanwoo cattle using DGEBV (upper panel), GEBV (middle panel), and residual (lower panel) as predictors in the GBLUP method. Q–Q plot showing the late separation between observed and expected *p*-values (-$\log_{10}$P). The red line represents the 95% concentration band under the null hypothesis of no association.

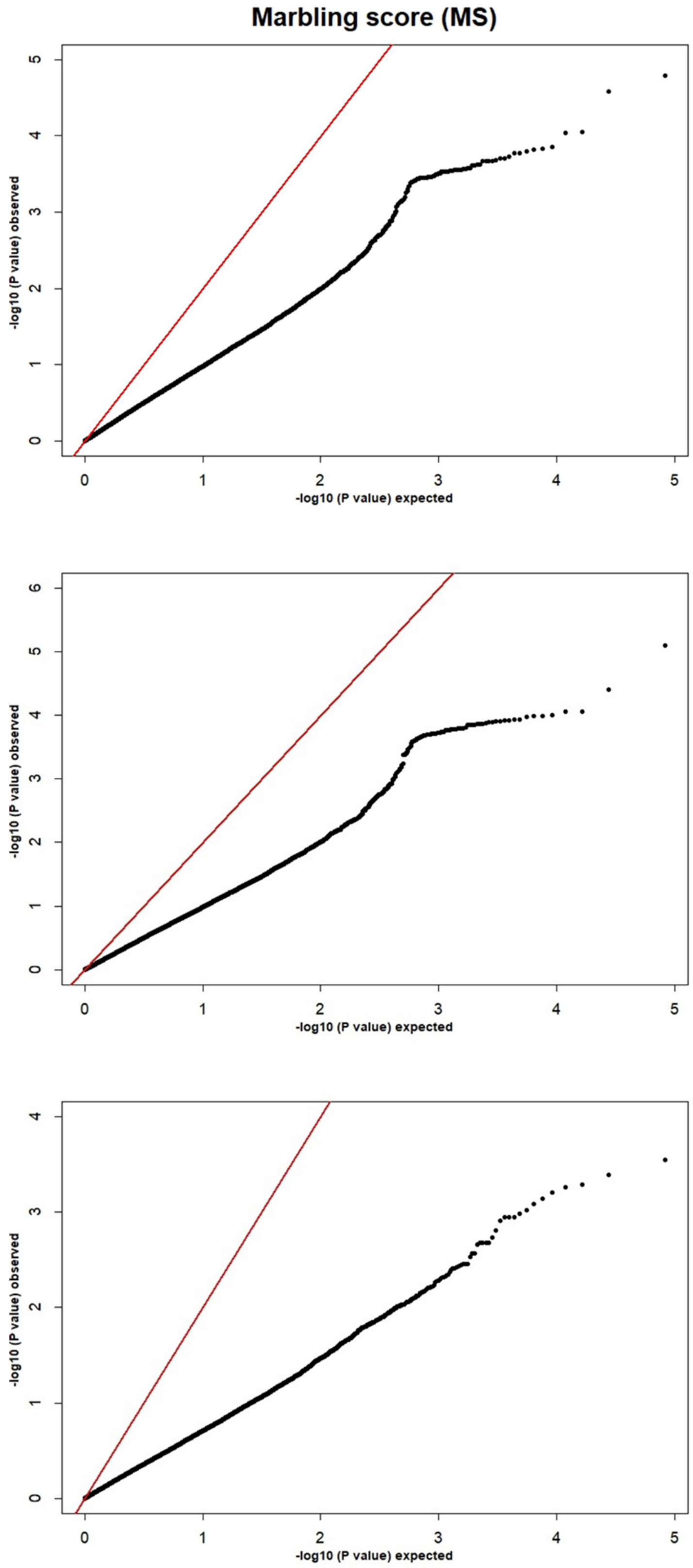

Figure S4. Quantile-quantile (Q-Q) plots for marbling score in Hanwoo cattle using DGEBV (upper panel), GEBV (middle panel), and residual (lower panel) as predictors in the GBLUP method. Q–Q plot showing the late separation between observed and expected *p*-values (-$\log_{10}$P). The red line represents the 95% concentration band under the null hypothesis of no association.

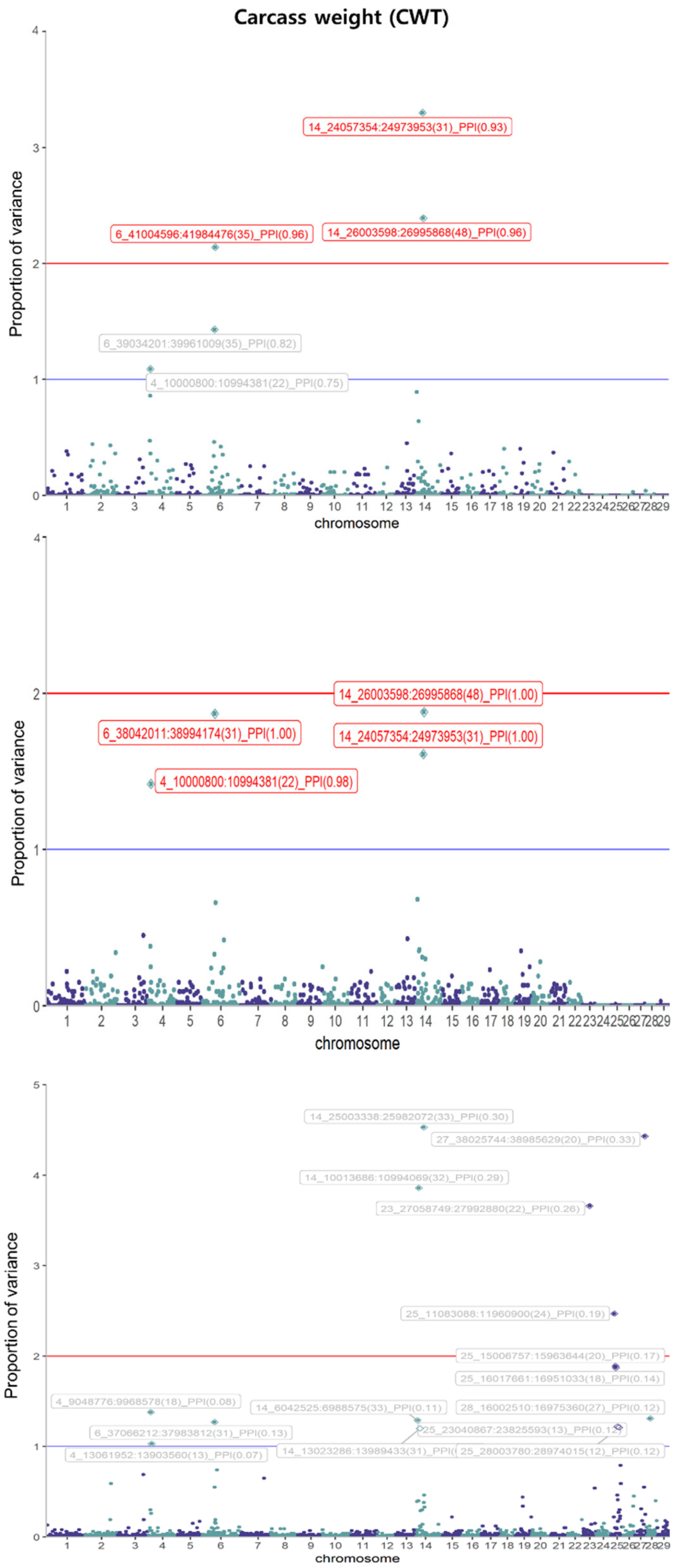

Figure S5. Genome-wide proportion of variance for carcass weight in Hanwoo cattle using DGEBV (upper panel), GEBV (middle panel), and residual (lower panel) as predictors in the Bayes B method. The X-axis represents the 29 *Bos taurus* autosomes, and the Y-axis represents the proportion of variance. The horizontal red line is the threshold 2.0 for significantly of windows.

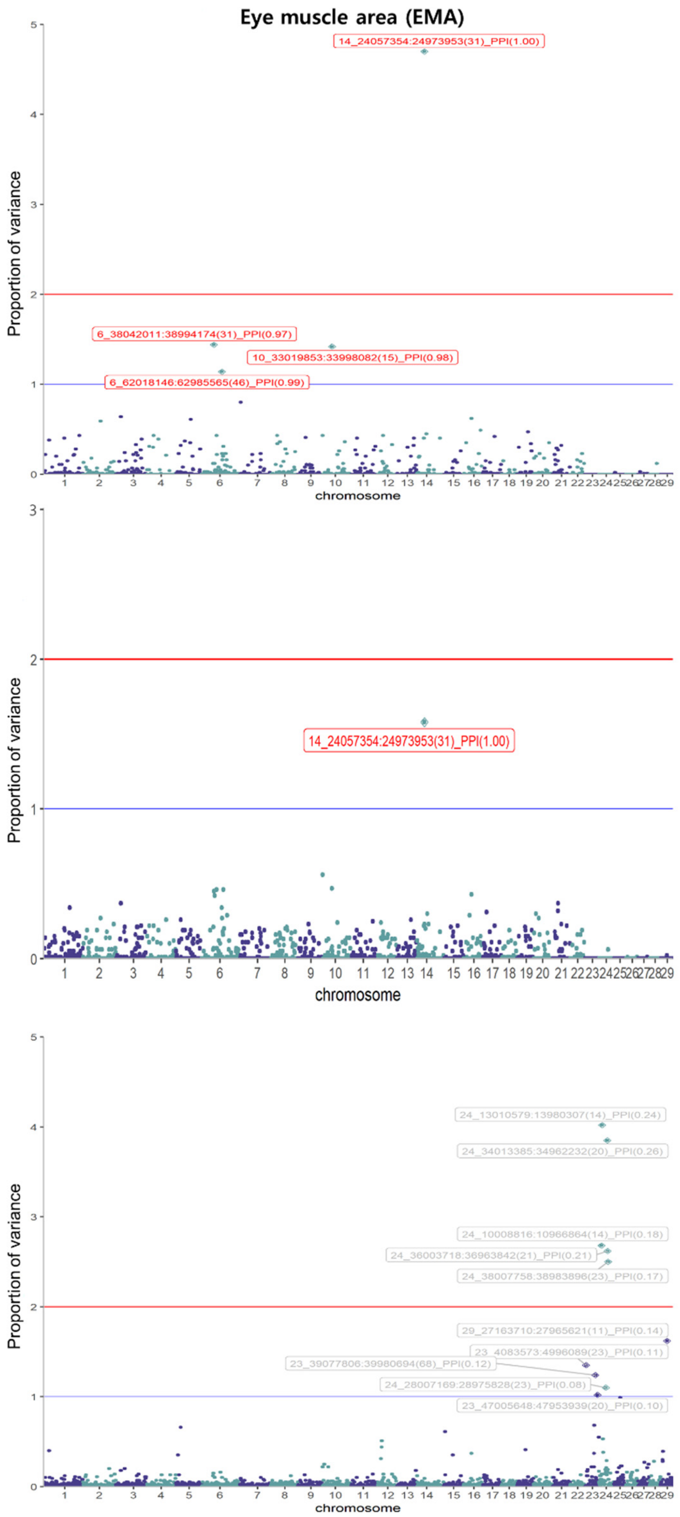

Figure S6. Genome-wide proportion of variance for eye muscle area in Hanwoo cattle using DGEBV (upper panel), GEBV (middle panel), and residual (lower panel) as predictors in the Bayes B method. The X-axis represents the 29 *Bos taurus* autosomes, and the Y-axis represents the proportion of variance. The horizontal red line is the threshold 2.0 for significantly of windows.

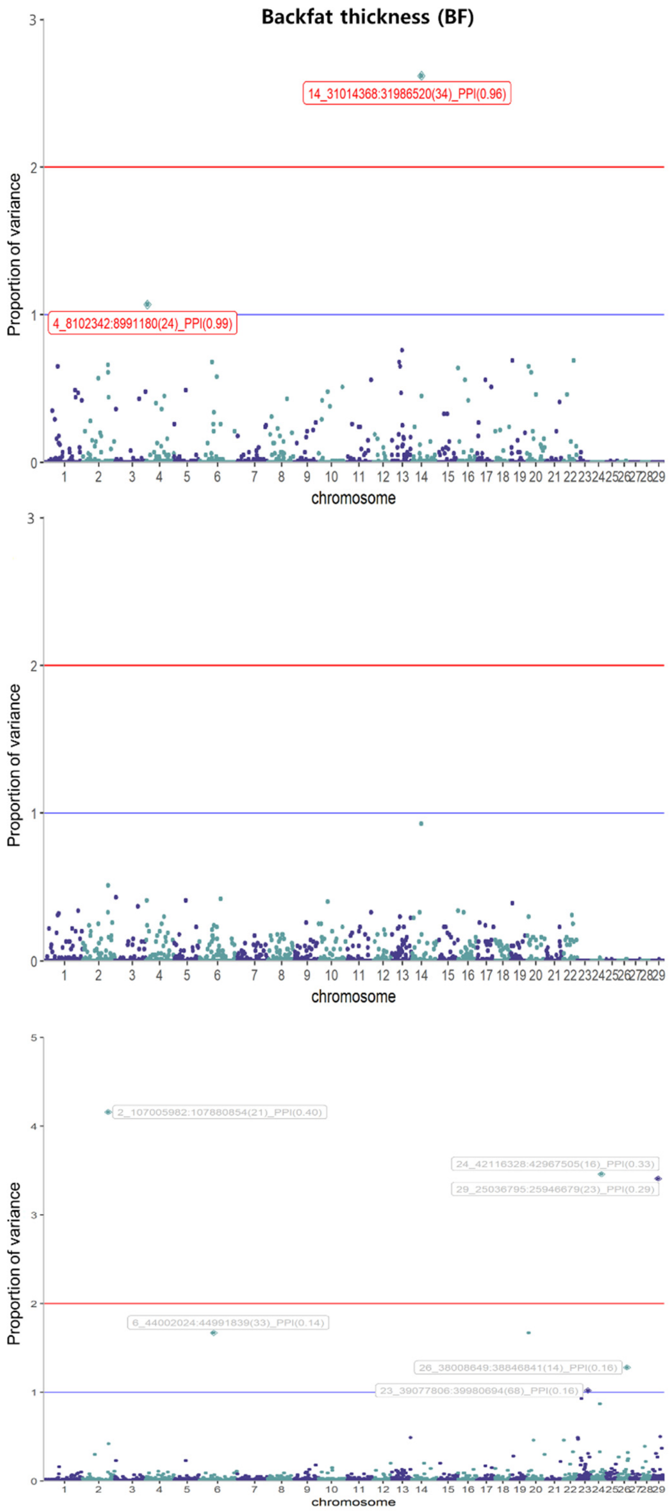

Figure S7. Genome-wide proportion of variance for backfat thickness in Hanwoo cattle using DGEBV (upper panel), GEBV (middle panel), and residual (lower panel) as predictors in the Bayes B method. The X-axis represents the 29 *Bos taurus* autosomes, and the Y-axis represents the proportion of variance. The horizontal red line is the threshold 2.0 for significantly of windows.

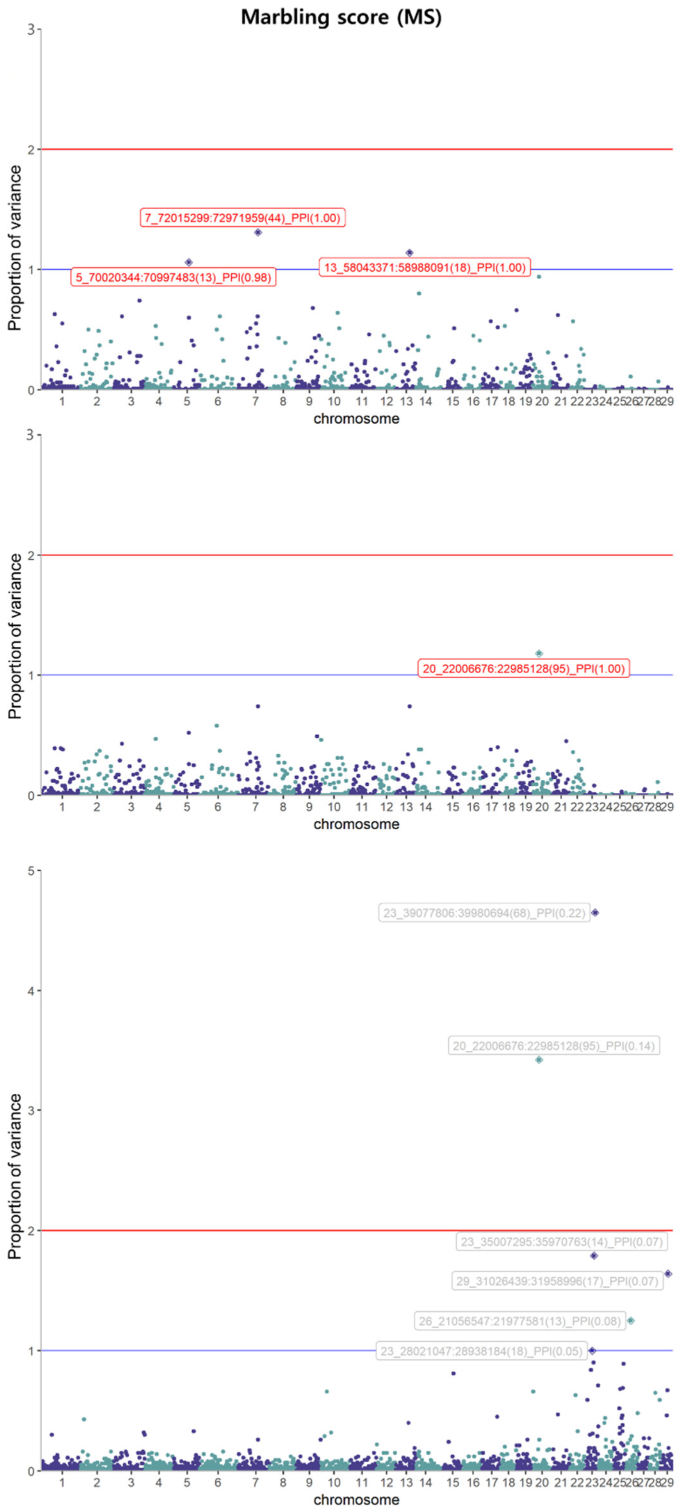

Figure S8. Genome-wide proportion of variance for marbling score in Hanwoo cattle using DGEBV (upper panel), GEBV (middle panel), and residual (lower panel) as predictors in the Bayes B method. The X-axis represents the 29 *Bos taurus* autosomes, and the Y-axis represents the proportion of variance. The horizontal red line is the threshold 2.0 for significantly of windows.

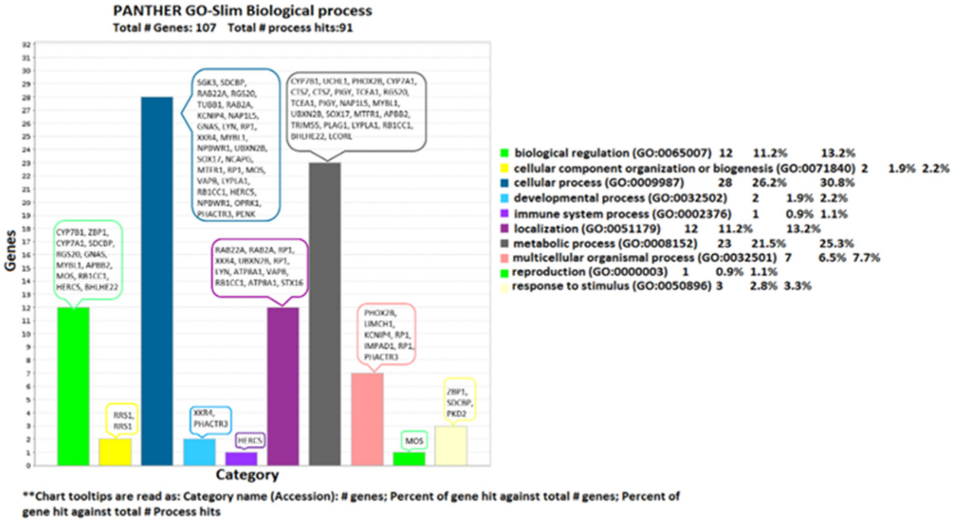

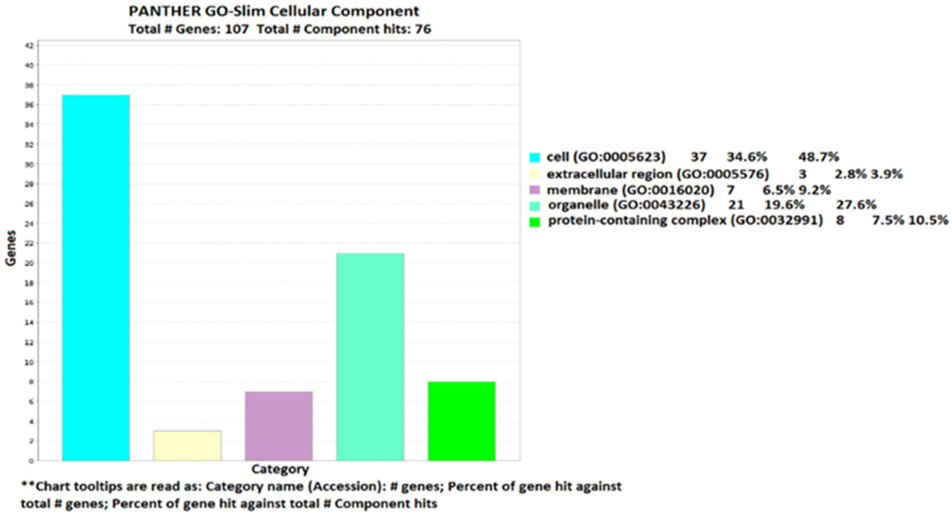

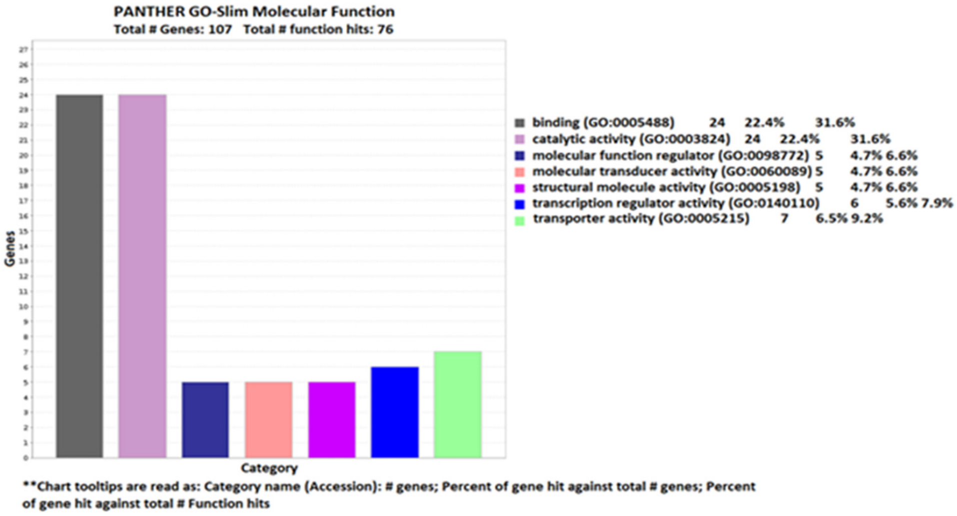

Figure S9. The PANTHER GO (Biological process, cellular components, and molecular function) is based on 107 selected genes from GWAS study, showing the percentage of gene hit against total process hits.

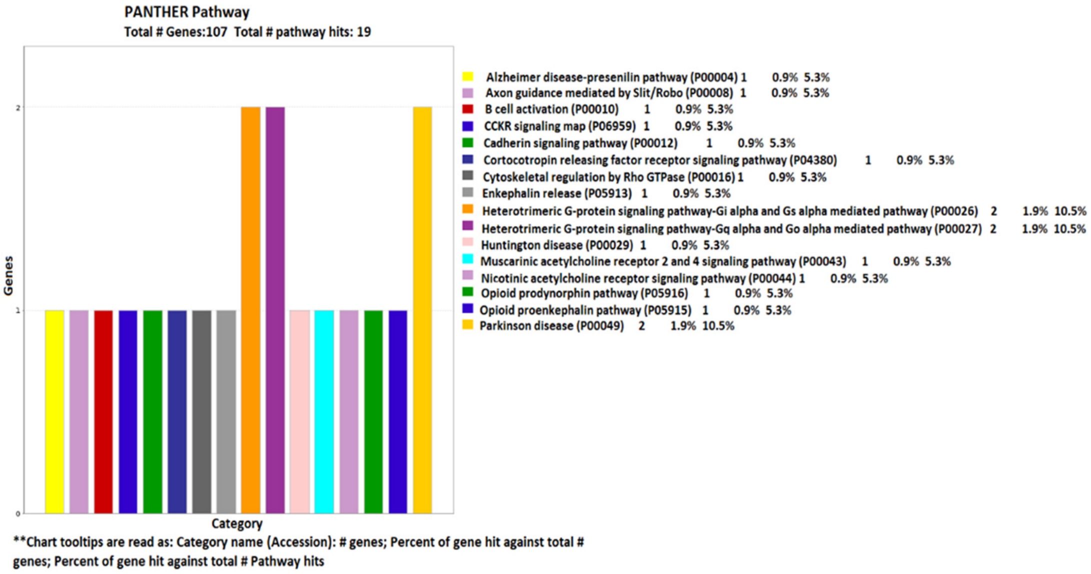

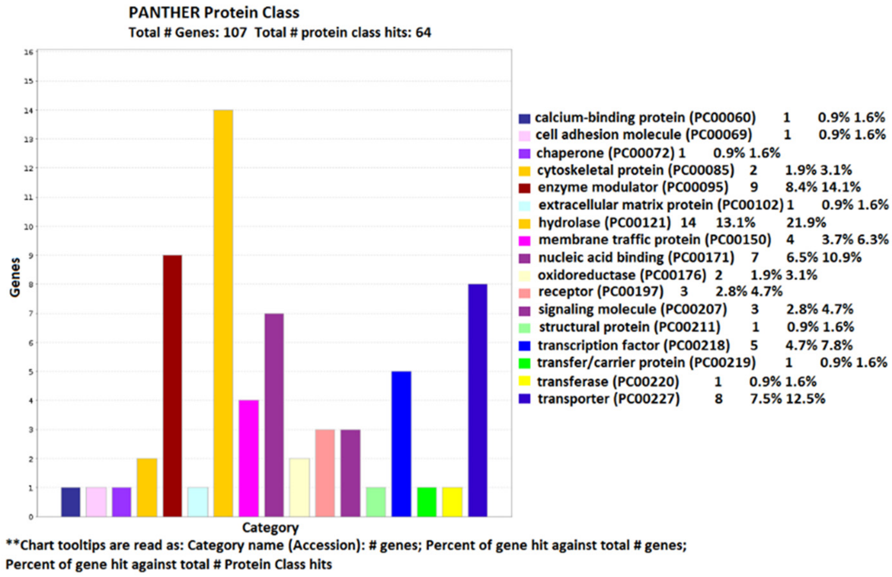

Figure S10. The PANTHER pathway (top) and protein class (down) based on 107 selected genes from GWAS study, showing the percent of gene hit against total process hits.

Table S1. Estimates of genetic variance, residual variance, phenotypic variance, and heritability for four carcass traits in Hanwoo using 5-fold cross validation.

Method	Traits	σ_a^2	σ_e^2	σ_p^2	h^2
GBLUP	CWT	703.18	919.18	1622.36	0.43
	EMA	43.05	73.81	116.86	0.37
	BF	7.89	13.97	21.86	0.36
	MS	1.21	1.34	2.55	0.47
Bayes B	CWT	820.18	1746.92	926.73	0.47
	EMA	43.19	118.34	75.15	0.36
	BF	8.08	22.01	13.93	0.37
	MS	1.06	2.48	1.42	0.43

σ_a^2, genetic variance; σ_e^2, residual variance; σ_p^2, phenotypic variance; h^2, heritability; GBLUP, genomic best linear unbiased prediction.

Table S2. Genome-wide significant common SNPs identified by GBLUP and Bayes B method, underlying CWT, EMA, BF, and MS traits in Hanwoo using de-regressed genomic estimated breeding values.

SNP	BTA: Position (bp)	Estimate ±SE	$-\log_{10}P$	Nearest gene	Consequence Type
**Carcass weight**					
rs380287756	6:41182962	4.465±0.136	8.534	*LOC782905, SLIT2*	IntGV
rs720848149	6:41262050	4.440±0.136	8.462	*SLIT2*	IV
rs470479052	6:41292118	4.120±0.122	9.024	*SLIT2*	IV
rs382125547	6:41311528	3.539±0.114	7.778	*SLIT2*	IV
rs384536472	6:41356212	3.562±0.114	7.784	*SLIT2*	IV
rs384017132	6:41438938	4.442±0.137	8.414	*SLIT2*	IV

rs719168867	6:41463763	4.467±0.137	8.508	*SLIT2*	IV
rs717602666	6:41526051	4.470±0.137	8.514	*SLIT2*	IV
rs721927483	6:41662240	4.458±0.136	8.516	*SLIT2, PACRGL*	IntGV
6:41957455	6:41957455	4.359±0.136	8.241	-	-
rs472474214	6:41984476	4.439±0.138	8.292	*KCNIP4*	IV
rs208411640	14:24141910	3.550±0.100	9.910	*RP1, XKR4*	IntGV
rs207487600	14:24266976	3.553±0.100	9.936	*RP1, XKR4*	IntGV
rs211204541	14:24332803	3.533±0.100	9.85	-	-
rs210090325	14:24343693	3.535±0.101	9.682	*XKR4*	IV
rs380287756	14:24391802	2.484±0.086	6.786	*XKR4*	IV
rs720848149	14:24432331	1.754±0.073	4.935	*XKR4*	IV
rs470479052	14:24437778	1.852±0.072	5.555	-	-
rs382125547	14:24462537	2.296±0.077	7.124	*XKR4*	IV
rs719168867	14:24891324	1.602±0.073	4.211	*LYN*	IV
rs717602666	14:24954981	1.862±0.078	4.885	*RPS20*	DGV
6:41957455	14:26023215	1.523±0.071	4.009	*LOC107133115*	IV
rs472474214	14:26083558	1.845±0.074	5.270	*FAM110B*	IV
rs208411640	14:26162492	3.603±0.089	12.366	*FAM110B, LOC101902490*	IntGV
rs207487600	14:26173037	3.607±0.089	12.230	*FAM110B, LOC101902490*	IntGV
rs211204541	14:26196375	3.433±0.088	11.785	*LOC101902490, UBXN2B*	IntGV
rs210090325	14:26223753	3.510±0.089	12.173	*LOC101902490, UBXN2B*	IntGV
rs42648867	14:26264142	3.509±0.089	12.408	*LOC101902490, UBXN2B*	IntGV
rs42649775	14:26351959	3.396±0.087	11.819	*TRNAG-CCC*	DGV
rs42649781	14:26395314	2.585±0.083	7.840	*CYP7A1, SDCBP*	IntGV
rs210258477	14:26399671	2.249±0.080	6.444	*CYP7A1, SDCBP*	IntGV
rs137279489	6:41182962	4.465±0.136	8.534	*LOC782905, SLIT2*	IntGV
rs108949025	6:41262050	4.440±0.136	8.462	*SLIT2*	IV
rs110092040	6:41292118	4.120±0.122	9.024	*SLIT2*	IV
rs42306838	6:41311528	3.539±0.114	7.778	*SLIT2*	IV
rs42305732	6:41356212	3.562±0.114	7.784	*SLIT2*	IV
rs42304778	6:41438938	4.442±0.137	8.414	*SLIT2*	IV
rs42304792	6:41463763	4.467±0.137	8.508	*SLIT2*	IV
rs42304759	6:41526051	4.470±0.137	8.514	*SLIT2*	IV
rs110634307	6:41662240	4.458±0.136	8.516	*SLIT2, PACRGL*	IntGV
rs42303720	6:41957455	4.359±0.136	8.241	-	-
rs43083563	6:41984476	4.439±0.138	8.292	*KCNIP4*	IV
rs41614868	14:24141910	3.550±0.100	9.910	*RP1, XKR4*	IntGV
rs210731567	14:24266976	3.553±0.100	9.936	*RP1, XKR4*	IntGV
rs41720580	14:24332803	3.533±0.100	9.850	-	-
rs209439851	14:24343693	3.535±0.101	9.682	*XKR4*	IV
rs41720533	14:26457030	2.237±0.080	6.357	-	-
rs41726059	14:26473490	2.141±0.080	5.984	*LOC101902713*	DGV
rs41725705	14:26570145	3.583±0.088	12.167	*NSMAF, LOC107133116*	IntGV
rs109374728	14:26656398	3.524±0.088	12.171	*TOX*	IV
rs41724548	14:26743126	3.595±0.088	12.181	*TOX*	IV
rs41724547	14:26746062	3.593±0.088	12.307	*TOX*	IV
rs41724536	14:26766010	3.607±0.089	12.324	-	-
rs41724028	14:26776546	3.592±0.088	12.335	*TOX*	IV
rs41627959	14:26804892	3.595±0.088	12.347	*TOX*	IV
rs208481177	14:26995868	3.184±0.085	10.979	-	-
**Eye muscle area**					
rs110578126	6:38373128	3.334±0.124	6.000	*IBSP, LOC104972726*	IntGV
rs110668054	6:38464203	0.534±0.023	4.917		
rs110983998	6:38477781	0.535±0.023	4.927	*LOC104972726, TRNAA-CGC*	IntGV
rs383197792	6:38509720	0.531±0.023	4.864	*LOC104972726, TRNAA-CGC*	IntGV
rs110857755	6:38632439	3.351±0.122	6.229	-	-
rs109438687	6:38648218	0.596±0.024	5.517	*FAM184B*	IV
rs110766531	6:38670165	0.597±0.024	5.562	-	-
rs136600360	6:38698689	0.570±0.023	5.324	-	-
rs110242144	6:38733885	0.600±0.024	5.587	*FAM184B*	IV
rs110175987	6:38783305	0.597±0.024	5.559	*NCAPG*	IV
rs379449143	6:38815034	0.597±0.024	5.559	-	-
rs109315734	6:38960730	0.598±0.024	5.579	*LCORL*	IV

rs109092727	6:38994174	0.456±0.022	4.032	*LCORL*	UGV
rs382379503	6:62615288	0.527±0.021	5.580	*SLC30A9, BEND4*	IV
rs208411640	14:24141910	0.449±0.020	4.637	*RP1, XKR4*	IV
rs207487600	14:24266976	0.447±0.020	4.602	*RP1, XKR4*	IV
rs211204541	14:24332803	0.449±0.020	4.633	-	-
rs210090325	14:24343693	0.445±0.020	4.514	*XKR4*	IV
rs42648867	14:24391802	0.350±0.017	4.054	*XKR4*	IV
rs134315607	14:24432331	1.754±0.073	4.935	*XKR4*	IV
rs42649775	14:24437778	1.852±0.072	5.555	-	-
rs42649781	14:24462537	0.352±0.015	4.942	*XKR4*	IV
rs137279489	14:24891324	1.602±0.073	4.211	*LYN*	IV
rs133832329	14:24920882	0.324±0.014	4.789	*LYN*	DGV
rs108949025	14:24954981	0.337±0.015	4.724	*RPS20*	DGV
rs110092040	14:24973953	0.499±0.017	7.019	*MOS*	DGV
rs42304778	14:26162492	0.506±0.018	7.062	*FAM110B, LOC101902490*	IntGV
rs42304792	14:26173037	0.507±0.018	7.076	*FAM110B, LOC101902490*	IntGV
rs42304759	14:26196375	0.495±0.017	6.976	*LOC101902490, UBXN2B*	IntGV
rs110634307	14:26223753	0.491±0.017	6.759	*LOC101902490, UBXN2B*	IntGV
rs42303720	14:26264142	0.491±0.017	6.759	*LOC101902490, UBXN2B*	IntGV
rs43083563	14:26311106	0.495±0.017	6.843	*UBXN2B*	DGV
rs41614868	14:26351959	0.483±0.017	6.807	*TRNAG-CCC*	DGV
rs210731567	14:26395314	0.346±0.016	4.192	*CYP7A1, SDCBP*	IntGV
rs41725705	14:26570145	0.504±0.017	7.083	*NSMAF, LOC107133116*	IntGV
rs41725159	14:26619895	0.503±0.017	7.076	*LOC107133116*	DGV
rs41725162	14:26621673	0.503±0.017	7.070	*LOC107133116*	DGV
rs109374728	14:26656398	0.495±0.017	6.898	*TOX*	IV
rs41724536	14:26766010	0.505±0.018	7.077	*TOX*	IV
rs41627960	14:26800529	0.292±0.014	4.015	*TOX*	IV
rs41627959	14:26804892	0.504±0.017	7.077	*TOX*	IV
rs42406063	14:26836013	0.293±0.014	4.037	*TOX*	IV
rs42406058	14:26848418	0.503±0.018	7.052	*TOX*	IV
rs42406039	14:26859737	0.504±0.017	7.077	*TOX*	IV
rs42404941	14:26967218	0.373±0.016	5.101	*TOX, CA8*	IntGV
rs208481177	14:26995868	0.431±0.017	5.778	-	-
**Backfat thickness**					
rs29021868	14:31014368	0.391±0.015	5.504	*CYP7B1*	IV
rs109003517	14:31144539	2.684±0.108	5.185	-	-
rs41613423	14:31197593	1.839±0.074	5.206	*LOC104974032, ARMC1*	IntGV
rs110811917	14:31447238	0.540±0.022	5.075	-	-
rs110684835	14:31616256	0.539±0.022	5.065	*LOC104974032, ARMC1*	IntGV
rs109048288	14:31646002	0.538±0.022	5.046	*LOC104974032, ARMC1*	IntGV
rs109858509	14:31684076	0.521±0.020	5.609	*LOC104974032, ARMC1*	IntGV
rs109658462	14:31795024	0.523±0.020	5.520	-	-
rs110141788	14:31820412	0.518±0.020	5.585	*PDE7A*	IV
rs109864374	14:31851268	0.518±0.020	5.585	*PDE7A*	IV
rs110055514	14:31986520	1.713±0.075	4.423	*PDE7A, LOC100299601*	IntGV
**Marbling score**					
rs110025998	13:58140449	-0.055±0.003	4.064	*LOC783163, C13H20orf85*	IV

SNP, single nucleotide polymorphism; BTA, *Bos taurus* autosome; bp, base pair (kb); SE, standard error; IntGV, intergenic variant; IV, intron variant; UGV, upstream gene variant; DGV, downstream gene variant.

Table S3. Genome-wide significant common SNPs identified by GBLUP and Bayes B method, underlying CWT, EMA, BF, and MS traits in Hanwoo using genomic estimated breeding values.

SNP	BTA: Position (bp)	Estimate±SE	-log$_{10}$P	Nearest gene	Consequence Type
**Carcass weight**					
rs110578126	6:38373128	17.200±0.634	6.079	*IBSP, LOC104972726*	IntGV
rs110668054	6:38464203	19.780±0.597	8.738	-	-
rs110983998	6:38477781	19.780±0.597	8.731	*LOC104972726, TRNAA-CGC*	IntGV
rs383197792	6:38509720	19.350±0.600	8.318	*LOC104972726, TRNAA-CGC*	IntGV

rs467005433	6:38530564	22.670±0.701	8.374	-	-
rs798880362	6:38580679	22.710±0.700	8.398	*LAP3*	IV
rs432843769	6:38581067	20.820±0.680	7.560	*LAP3*	IV
rs110857755	6:38632439	17.220±0.624	6.266	-	-
rs109438687	6:38648218	22.010±0.622	9.858	*FAM184B*	IV
rs110766531	6:38670165	22.060±0.621	9.952	-	-
rs136600360	6:38698689	20.930±0.608	9.372	-	-
rs110242144	6:38733885	22.110±0.621	9.976	*FAM184B*	IV
rs110175987	6:38783305	22.120±0.621	10.006	*NCAPG*	IV
rs379449143	6:38815034	22.120±0.621	10.006	-	-
rs110834363	6:38939012	17.360±0.571	7.474	*LCORL*	IV
rs109315734	6:38960730	22.120±0.621	10.004	*LCORL*	IV
rs109092727	6:38994174	17.530±0.573	7.559	*LCORL*	IV
rs208411640	14:24141910	18.720±0.510	10.539	*RP1, XKR4*	IntGV
rs209201458	14:24183559	8.962±0.411	4.118	*RP1, XKR4*	IntGV
rs210355704	14:24221973	8.981±0.411	4.131	-	-
rs207487600	14:24266976	18.730±0.511	10.549	*RP1, XKR4*	IntGV
rs211204541	14:24332803	18.650±0.510	10.505	-	-
rs210090325	14:24343693	18.660±0.515	10.325	*XKR4*	IV
rs42648867	14:24391802	13.040±0.440	7.128	*XKR4*	IV
rs134315607	14:24432331	9.221±0.371	5.185	*XKR4*	IV
rs42649775	14:24437778	9.700±0.367	5.799	-	-
rs42649781	14:24462537	12.170±0.395	7.633	*XKR4*	IV
rs210258477	14:24497726	18.540±0.446	13.277	-	-
rs42646659	14:24524210	18.570±0.447	13.328	*XKR4*	IV
rs137279489	14:24891324	8.467±0.371	4.460	*LYN*	IV
rs133832329	14:24920882	8.254±0.382	4.052	LYN	DGV
rs108949025	14:24954981	9.897±0.396	5.239	RPS20	DGV
rs42306838	14:26023215	8.177±0.363	4.371	*LOC107133115*	IV
rs42305732	14:26083558	9.806±0.376	5.657	*FAM110B*	IV
rs210731567	14:26395314	13.770±0.422	8.502	*CYP7A1, SDCBP*	IntGV
rs41720580	14:26399671	12.130±0.409	7.146	*CYP7A1, SDCBP*	IntGV
rs41720533	14:26457030	12.070±0.409	7.054	-	-
rs41726059	14:26473490	11.520±0.406	6.592	*LOC101902713*	DGV
rs41725159	14:26619895	18.980±0.449	13.724	*LOC107133116*	DGV
rs41725162	14:26621673	18.980±0.449	13.712	*LOC107133116*	DGV
rs109374728	14:26656398	18.670±0.449	13.287	*TOX*	IV
rs41724548	14:26743126	19.070±0.450	13.277	*TOX*	IV
rs41724547	14:26746062	19.060±0.449	13.254	*TOX*	IV
rs41724536	14:26766010	19.100±0.450	13.194	*TOX*	IV
rs41724028	14:26776546	19.050±0.449	13.188	*TOX*	IV
rs41627960	14:26800529	8.278±0.378	4.149	*TOX*	IV
rs41627959	14:26804892	19.070±0.449	13.161	*TOX*	IV
rs42406063	14:26836013	8.325±0.378	4.197	*TOX*	IV
rs42406058	14:26848418	19.060±0.450	13.159	*TOX*	IV
rs42406039	14:26859737	19.070±0.449	12.763	*TOX*	IV
rs42404949	14:26949215	8.156±0.370	4.197	*TOX, CA8*	IntGV
rs42404941	14:26967218	16.280±0.396	12.690	*TOX, CA8*	IntGV
rs208481177	14:26995868	16.910±0.432	11.896	-	-
**Eye muscle area**					
rs110668054	6:38464203	3.252±0.135	5.108	-	-
rs110983998	6:38477781	3.254±0.135	5.113	*LOC104972726, TRNAA-CGC*	IntGV
rs383197792	6:38509720	3.226±0.135	5.030	*LOC104972726, TRNAA-CGC*	IntGV
rs109438687	6:38648218	3.642±0.141	5.765	*FAM184B*	IV
rs110766531	6:38670165	3.647±0.141	5.809	-	-
rs136600360	6:38698689	3.462±0.137	5.508	-	-
rs110242144	6:38733885	3.660±0.141	5.831	*FAM184B*	IV
rs110175987	6:38783305	3.647±0.141	5.806	*NCAPG*	IV
rs379449143	6:38815034	3.647±0.141	5.806	-	-
rs110834363	6:38939012	2.754±0.129	4.125	*LCORL*	IV
rs109315734	6:38960730	3.651±0.140	5.823	*LCORL*	IV
rs109092727	6:38994174	2.776±0.129	4.175	*LCORL*	UGV
rs208411640	14:24141910	2.758±0.117	4.893	*RP1, XKR4*	IV
rs209201458	14:24183559	8.962±0.411	4.118	*RP1, XKR4*	IntGV

rs210355704	14:24221973	8.981±0.411	4.131	-	-
rs207487600	14:24266976	2.750±0.117	4.862	*RP1, XKR4*	IV
rs211204541	14:24332803	2.760±0.117	4.904	-	-
rs210090325	14:24343693	2.740±0.118	4.781	*XKR4*	IV
rs42648867	14:24391802	2.134±0.098	4.208	*XKR4*	IV
rs134315607	14:24432331	9.221±0.371	5.185	*XKR4*	IV
rs42649775	14:24437778	9.700±0.367	5.799	-	-
rs42649781	14:24462537	2.165±0.088	5.245	*XKR4*	IV
rs137279489	14:24891324	8.467±0.371	4.460	*LYN*	IV
rs133832329	14:24920882	1.993±0.083	5.055	*LYN*	DGV
rs108949025	14:24954981	2.063±0.087	4.954	*RPS20*	DGV
rs110092040	14:24973953	3.069±0.103	7.469	*MOS*	DGV
rs42305732	14:26083558	1.820±0.083	4.287	*FAM110B*	IV
rs42304759	14:26196375	3.053±0.102	7.452	*LOC101902490, UBXN2B*	IntGV
rs110634307	14:26223753	3.023±0.103	7.190	*LOC101902490, UBXN2B*	IntGV
rs42303720	14:26264142	3.023±0.103	7.200	*LOC101902490, UBXN2B*	IntGV
rs43083563	14:26311106	3.047±0.103	7.292	*UBXN2B*	DGV
rs41614868	14:26351959	2.973±0.101	7.240	*TRNAG-CCC*	DGV
rs210731567	14:26395314	2.140±0.095	4.482	*CYP7A1, SDCBP*	IntGV
rs41720580	14:26399671	1.948±0.092	4.068	*CYP7A1, SDCBP*	IntGV
rs41720533	14:26457030	1.941±0.092	4.038	-	-
rs41725162	14:26621673	3.096±0.103	7.526	*LOC107133116*	DGV
rs109374728	14:26656398	3.039±0.103	7.308	*TOX*	IV
rs41724536	14:26766010	3.108±0.104	7.523	*TOX*	IV
rs41627960	14:26800529	1.794±0.083	4.220	*TOX*	IV
rs41627959	14:26804892	3.101±0.103	7.533	*TOX*	IV
rs42406063	14:26836013	1.798±0.082	4.247	*TOX*	IV
rs42406058	14:26848418	3.099±0.103	7.507	*TOX*	IV
rs42406039	14:26859737	3.101±0.103	7.533	*TOX*	IV
rs42404941	14:26967218	2.290±0.092	5.371	*TOX, CA8*	IntGV
rs208481177	14:26995868	2.652±0.099	6.132	-	-

SNP, single nucleotide polymorphism; BTA, *Bos taurus* autosome; bp, base pair (kb); SE, standard error; IntGV, intergenic variant; IV, intron variant; DGV, downstream gene variant.

Table S4. Functional cluster enrichment analysis of gene via DAVID database for the carcass traits in Hanwoo.

Term	Genes count	%	*p*-value	FDR
**Annotation Cluster 1**	**Enrichment Score: 1.6400**			
IPR000408:Regulator of chromosome condensation, RCC1	3	3.16	0.004	4.70
IPR009091:Regulator of chromosome condensation	3	3.16	0.005	5.70

1/beta-lactamase-inhibitor protein II				
SM00119:HECTc	3	3.16	0.006	5.56
IPR000569:HECT	3	3.16	0.007	7.96
Cell division and chromosome partitioning / Cytoskeleton	3	3.16	0.008	3.87
GO:0016874~ligase activity	4	4.21	0.011	12.53
Ubl conjugation pathway	5	5.26	0.040	36.99
GO:0042787~protein ubiquitination involved in ubiquitin-dependent protein catabolic process	3	3.16	0.116	82.14
GO:0004842~ubiquitin-protein transferase activity	3	3.16	0.239	95.96
Transferase	6	6.32	0.619	100.00
**Annotation Cluster 2**		**Enrichment Score: 0.6602**		
GO:0003924~GTPase activity	4	4.21	0.055	48.71
GO:0005525~GTP binding	4	4.21	0.245	96.28
IPR027417:P-loop containing nucleoside triphosphate hydrolase	4	4.21	0.771	100.00
**Annotation Cluster 3**		**Enrichment Score: 0.5895**		
Mitochondrion	6	6.32	0.23	94.67
Transit peptide	4	4.21	0.23	95.00
transit peptide:Mitochondrion	4	4.21	0.32	98.84
**Annotation Cluster 4**		**Enrichment Score: 0.5081**		
GO:0007268~chemical synaptic transmission	4	4.21	0.02	24.40
GO:0007218~neuropeptide signaling pathway	3	3.16	0.05	51.24
Glycoprotein	11	11.58	0.06	51.99
topological domain:Extracellular	5	5.26	0.42	99.79
topological domain:Cytoplasmic	6	6.32	0.50	99.96
glycosylation site:N-linked (GlcNAc...)	7	7.37	0.57	99.99
transmembrane region	8	8.42	0.67	100.00
disulfide bond	5	5.26	0.76	100.00
Cell membrane	7	7.37	0.81	100.00
GO:0005887~integral component of plasma membrane	3	3.16	0.91	100.00
Disulfide bond	6	6.32	0.92	100.00
**Annotation Cluster 5**		**Enrichment Score: 0.2575**		

GO:0005615~extracellular space	7	7.37	0.31	98.5
Secreted	6	6.32	0.44	99.9
Signal	13	13.68	0.77	100.0
signal peptide	5	5.26	0.88	
**Annotation Cluster 6**	**Enrichment Score: 0.1981**			
Nucleotide-binding	7	7.37	0.49	99.96
IPR017441:Protein kinase, ATP binding site	3	3.16	0.51	99.99
ATP-binding	5	5.26	0.62	100.00
Transferase	6	6.32	0.62	100.00
IPR000719:Protein kinase, catalytic domain	3	3.16	0.66	100.00
Kinase	3	3.16	0.66	100.00
IPR011009:Protein kinase-like domain	3	3.16	0.71	100.00
GO:0005524~ATP binding	5	5.26	0.87	100.00
**Annotation Cluster 7**	**Enrichment Score: 0.0606**			
transmembrane region	8	8.42	0.67	100.00
Membrane	25	26.32	0.86	100.00
GO:0016021~integral component of membrane	17	17.89	0.90	100.00
Transmembrane helix	18	18.95	0.98	100.00
Transmembrane	18	18.95	0.98	100.00
